# Construction of transplantable artificial vascular tissue based on adipose tissue-derived mesenchymal stromal cells by a cell coating and cryopreservation technique

**DOI:** 10.1038/s41598-021-97547-2

**Published:** 2021-09-09

**Authors:** Yoshiya Asano, Daisuke Okano, Michiya Matsusaki, Tetsuro Watabe, Yasuhiro Yoshimatsu, Mitsuru Akashi, Hiroshi Shimoda

**Affiliations:** 1 Department of Neuroanatomy, Cell Biology and Histology, Graduate School of Medicine, Hirosaki University, Hirosaki, Japan; 2 Department of Applied Chemistry, Graduate School of Engineering, Osaka University, Osaka, Japan; 3 Department of Biochemistry, Graduate School of Medical and Dental Sciences, Tokyo Medical and Dental University (TMDU), Tokyo, Japan; 4 Division of Pharmacology, Graduate School of Medical and Dental Sciences, Niigata University, Niigata, Japan; 5 Building Block Science, Graduate School of Frontier Biosciences, Osaka University, Osaka, Japan; 6 Department of Anatomical Science, Graduate School of Medicine, Hirosaki University, Hirosaki, Japan

**Keywords:** Regenerative medicine, Tissue engineering

## Abstract

Prevascularized artificial three-dimensional (3D) tissues are effective biomaterials for regenerative medicine. We have previously established a scaffold-free 3D artificial vascular tissue from normal human dermal fibroblasts (NHDFs) and umbilical vein-derived endothelial cells (HUVECs) by layer-by-layer cell coating technique. In this study, we constructed an artificial vascular tissue constructed by human adipose tissue-derived stromal cells (hASCs) and HUVECs (ASCVT) by a modified technique with cryopreservation. ASCVT showed a higher thickness with more dense vascular networks than the 3D tissue based on NHDFs. Correspondingly, 3D-cultured ASCs showed higher expression of several angiogenesis-related factors, including vascular endothelial growth factor-A and hepatic growth factor, compared to that of NHDFs. Moreover, perivascular cells in ASCVT were detected by pericyte markers, suggesting the differentiation of hASCs into pericyte-like cells. Subcutaneous transplantation of ASCVTs to nude mice resulted in an engraftment with anastomosis of host’s vascular structures at 2 weeks after operation. In the engrafted tissue, the vascular network was surrounded by mural-like structure-forming hASCs, in which some parts developed to form vein-like structures at 4 weeks, suggesting the generation of functional vessel networks. These results demonstrated that cryopreserved human cells, including hASCs, could be used directly to construct the artificial transplantable tissue for regenerative medicine.

## Introduction

Regenerative medicine by transplantation of artificial tissue is an ideal alternative to organ transplantation or when the treatment cannot be accomplished by usual medicine. Scaffold-free fabrication of artificial tissue by human cells is a trend in regenerative medicine regarding its low antigenicity and safety in transplantation and engraftment^1,2^. Moreover, prevascularization of the artificial tissues has been attempted for medical applications^3–5^, since blood circulation is critical for an effective engraftment of artificial tissues in order to prevent tissue degeneration due to low supply of nutrition and oxygen^3,6^. However, their application is still under development due to high costs, complication in fabrication, bio safety in human transplantation, and lack of well-established long-term grafts with vascular functions^7^.

In our previous studies, three-dimensional (3D) artificial tissue with blood/lymphatic vascular network has been established using cell accumulation method with an extracellular matrix (ECM)-based layer-by-layer cell coating technique^8,9^. In this technique, the cultured human cells are coated with fibronectin and gelatin layers, resulting in a 10 nm-thick coating ECM nano-films. Although these cells are free from artificial scaffolds, such as fibrin gel, they can form 3D artificial tissue within a short-term. For fabrication of vascular tissue, the fibronectin and gelatin-coated endothelial cells are cultivated between the layers of normal human dermal fibroblasts (NHDFs). They rapidly form a network of blood and/or lymphatic vessel-like tubules within a week^9,10^. To confirm their function, we have transplanted the 3D tissues with vascular networks, which were constructed using NHDFs and human umbilical vein-derived endothelial cells (HUVECs), into the subcutaneous tissue of nude mice. We found the engraftment of artificial vasculatures accompanied with their anastomosis to the host blood vessels and inflow of blood^3^. Moreover, the lymphatic vascular tissue constructed using NHDFs and human dermal lymphatic endothelial cells showed remodeling of the network with generation of blood vessel-like structures after engraftment into the back fascia or subcutaneous tissue of nude mice^4^. From these studies, we proposed the potency of the artificial vascular tissues constructed by the cell accumulation method in regenerative medicine^3,4^.

For the therapeutic application of artificial vascular tissues, use of autologous cells for transplantation is a reasonable strategy to avoid host rejection. Autologous mesenchymal stem/stromal cells (MSCs) are effective biomaterials for constructing tissue allowing successful engraftment^11^. In particular, adipose tissue-derived stromal cells (ASCs) are considered as an attractive biomaterial because the adipose tissue isolation from the donors or patients is minimally invasive. To date, much of studies for cellular transplantation of ASCs and their therapeutic applications have been reported, and it has been suggested that these cells induce tissue regeneration, differentiate to host tissue, and show immunomodulatory activities^12,13^. Because ASCs can induce blood and lymphatic vessel regeneration, these cells are suitable for the treatment of ischemic diseases and lymphedema^4,14^. For transplantation, there are also tissue engineering efforts using ASCs to construct cell sheets^15^ and 3D culture with artificial scaffolds^16,17^. Regarding the application of vascular tissue engineering, the co-cultivation of ASCs and endothelial cells in the fibrin matrix have been reported^18,19^. From these studies, the vascular tissue engineering will soon reach clinical applications. Scaffold-free strategies with simple and rapid technique would contribute to practical applications in regenerative medicine.

In the present study, we aimed to construct transplantable vascular tissue based on human ASCs (hASCs) by ECM-based cell accumulation method, with an addition of cryopreservation technique to elevate its availability in medical studies and clinical applications. For this purpose, the ECM nano-film-coated cells were cryopreserved using appropriate condition and used for tissue construction (refers as cryopreserved cell accumulation method; CP-CAM). By this method, we constructed the transplantable human vascular tissues based on hASCs, then evaluated in vitro characteristics and subcutaneous engraftment to nude mice, in comparison with those of fibroblast-based tissue as reported in our previous studies^3,9^.

## Results

### Vascular network formation in hASC-based 3D artificial vascular tissue fabricated by CP-CAM

In this study, we developed CP-CAM by combining cell accumulation method with cryopreservation technique. To optimize the conditions, we firstly examined the vessel network formation using artificial vascular tissue constructed by NHDFs and HUVECs, termed as FbVT (Supplementary information, Fig. S1–S4). Briefly, our results showed that ECM nano-film coated cells frozen by using CultureSure Freezing Medium (Fujifilm Wako, termed as CSFM in this study), containing dimethyl sulfoxide (DMSO) and albumin, can formed vessel network after thawing (Fig. S1e). We examined whether the albumin in CSFM is an effective substance for CP-CAM, by using our original freezing medium containing recombinant human albumin (refers to DMSO-albumin FM as described in “Methods”). The vessel network formation from the DMSO-albumin FM-frozen cells was found, suggesting an efficacy of albumin for this method (Fig. S1f). However, the higher yields of living cells after thawing of cryopreserved stocks were observed in CSFM (data not shown). Therefore, we continuously used CSFM for CP-CAM in this study. In comparison between FbVT with or without cryopreservation using CSFM, no differences were found in the vascular network structures (Supplementary information, Fig. S3).

**Figure 1:**
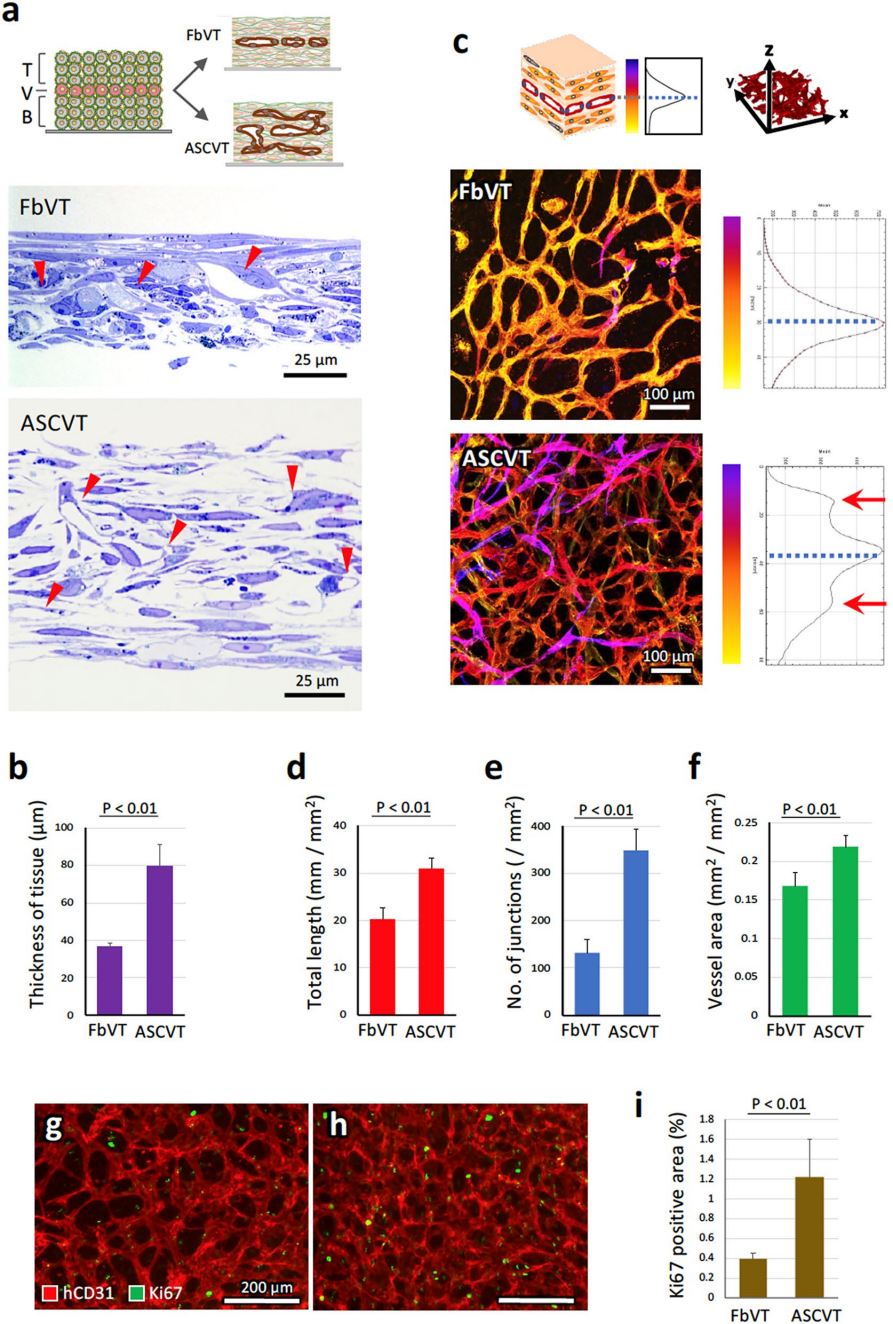
Comparison of the vascular tissues constructed by CP-CAM based on NHDFs and hASCs. (**a**) The vascular tissues were constructed by sequential pile of basal four layers of NHDF or hASC (B), middle single layer of HUVEC (V), and top four layers of NHDF or hASC (T). For 4 days, 3D cultivation of the piled cells resulted the formation of artificial tissues with vascular structures. The microscopic images of toluidine blue staining sections are shown (FbVT: vascular tissue based on NHDFs, ASCVT: vascular tissue based on hASCs). Arrowheads indicate the distribution of the vascular structures. (**b**) Comparison of the thickness of FbVT and ASCVT (N = 5). (**c**) The integrated confocal 3D images of human CD34-positive vascular networks in FbVT and ASCVT. Color bars denote the depth along z-axis of the tissue, and the vascular images colored according to the depth of the tissues. The graphs show the relative density distribution of the vascular structure along the depth of the FbVT and ASCVT. Hatched lines indicate the depth of middle vascular layer. The vascular network of ASCVT showed a more dense structure compared to that of FbVT, and spreads to the top and bottom layers indicated by multiple color of the vascular images. Accordingly, the additional peaks of vascular distribution appeared at top and bottom layers as indicated by red arrows. (**d**–**f**) Quantitative analysis of vascular total length (**d**), number of junctions (**e**), and vessel area (**f**) in FbVT and ASCVT (N = 6). (**g**–**i**) The cell proliferation in FbVT and ASCVT (4 days after the cell seeding) is evaluated by immunostaining for Ki67, a marker of cell cycle progression. (**g**,**h**) Histological images of FbVT (**g**) and ASCVT (**h**). The vascular structures are visualized by immunostaining for human CD31 (hCD31). Ki67-positive cells are more frequently observed in ASCVT. (**i**) Quantitative analysis indicates that Ki67-positive area in ASCVT is higher than that of FbVT (N = 5). The images are representatives from five independent experiments.

Next, we fabricated artificial vascular tissue constructed by hASCs and HUVECs, termed as ASCVT, by CP-CAM, and the tissue structure was compared with that of the FbVT. As illustrated in Fig. 1a, single layer of HUVECs was cultivated for 4 days between the bottom and top 4 layers of NHDFs or hASCs. The section of FbVT showed the distribution of vascular structure at the middle layer of the tissue (Fig. 1a, FbVT, arrowheads), similar to findings in previous reports^9,10^. On the other hand, ASCVT had abundant extracellular space between hASCs and showed about two times thicker than FbVT (Fig. 1a, ASCVT and Fig. 1b). The distribution of the vascular structure was not only in the middle part but also in the basal and top layer of the tissue (Fig. 1a, ASCVT, arrowheads). Three-dimensional analysis of the tissues clearly showed these distribution patterns of the vascular structure (Fig. 1c, FbVT and ASCVT); the color labeling of vascular structure in FbVT and ASCVT along the depth of the tissue demonstrated the distribution of the network at the middle layer of FbVT and that at the top, middle, and bottom layers of ASCVT. The vertical distribution of the vascular structure showed additional peaks at top and bottom layers (Fig. 1c, ASCVT, arrows) suggesting the three-dimensional elongation of the vessels in ASCVT. Furthermore, the vascular network density of ASCVT was higher than that of FbVT (Fig. 1c). Further quantitative analysis of vascular network structures at the middle plane of FbVT and ASCVT confirmed these findings. The results in Fig. 1d–f showed a significantly higher score for total length, number of junctions, and vessel area in ASCVT than FbVT.

To evaluate whether cell proliferation contributes to tissue thickness, the tissues were immunostained for Ki67, a marker of the cell proliferation, and the positive areas were compared between FbVT and ASCVT (Fig. 1g–i). The results showed that Ki67-positive areas in the ASCVT were more frequently observed, demonstrating a higher frequency of cell proliferation in this tissue. The Ki67-positive staining in the ASCVT was observed in the nuclei of both hASCs and HUVECs.

We also evaluated the influence of CP-CAM-based cryopreservation on ASCVT construction, since the recovery rate of coated and frozen hASCs was lower than that of the NHDFs (Supplementary information, Fig. S2). As shown in Fig. S5, there were no significant differences in the network structure and thickness of the ASCVTs constructed with or without freezing technique. This result demonstrated the usability of frozen hASCs for vascular tissue construction in this study.

### Analysis of angiogenesis-related factors profiles

**Figure 2:**
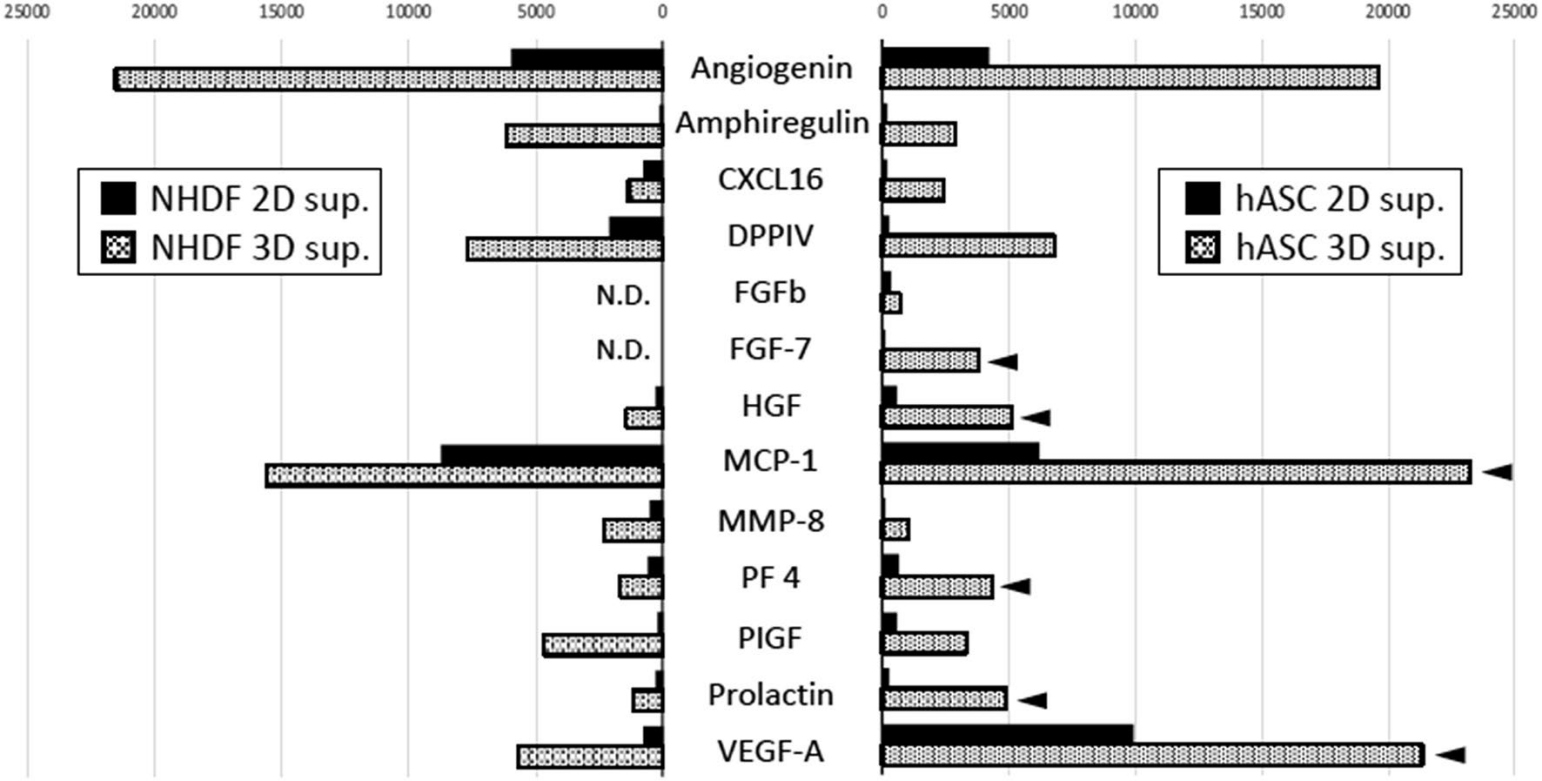
Profiles of angiogenesis-related factors in culture supernatants collected from 2D- and 3D-cultivated NHDFs and hASCs. The culture supernatants were analyzed using Proteome Profiler Human Angiogenesis Array Kit (R&D Systems). Among 55 angiogenesis-related factors, 13 factors increased in 3D cultivation compared to 2D cultivation of NHDFs or ASCs and are shown in these graphs (ND: not detected). Arrowheads: the factors specifically increased in the supernatant from 3D cultivation of ASCs.

The results above demonstrated that the pro-angiogenic/vasculogenic activity of hASCs is more potent than NHDFs in the 3D culture. To confirm the key factors, the angiogenesis-related factors in the culture supernatants of 2D/3D cultivated NHDFs and hASCs were examined by Proteome Profilar Human Angiogenesis Array Kit. We obtained the results of 55 factors as arrays of the dot immunoassays. Among them, the relative expression levels of thirteen factors were promoted in 3D cultivation compared to 2D cultivation as shown in Fig. 2. Three-dimensional culture of NHDFs showed an increased expression levels of eleven factors, including vascular endothelial growth factor-A (VEGF-A) and hepatocyte growth factor (HGF), as shown in our previous study^9^. In the present study, we detected increased amount of other factors including angiogenin^20^, amphiregulin^21^, and PIGF^22^, the mediators of endothelial cell migration and tubular structure formation such as CXCL16^23^, MCP-1^24^, MPP-8^25^, and prolactin^26^, and the angiogenic regulatory factors such as DPPIV^27^ and PF4^28^. The 3D culture of hASCs also showed a similar profile of an increased expression of the angiogenesis-related factors. However, in particular, six factors were intensely promoted as indicated by the arrowheads in Fig. 2. Among these factors, an increase expression of FGF-7 (a keratinocyte growth factor)^29^ was specifically found in the 3D culture of hASCs.

In the artificial tissue fabricated by cell accumulation method, hypoxic condition has been shown in the tissue microenvironment^9^. Hence, the increase of the factors in 3D cultivated hASCs might be due to the hypoxic microenvironment. Therefore, we further analyzed the profile in 2D-cultured hASCs prepared under hypoxic condition. As shown in Fig. S6, the profile did not correspond with those of 3D cultivations.

### Pericyte-like differentiation of perivascular hASCs in ASCVT

**Figure 3:**
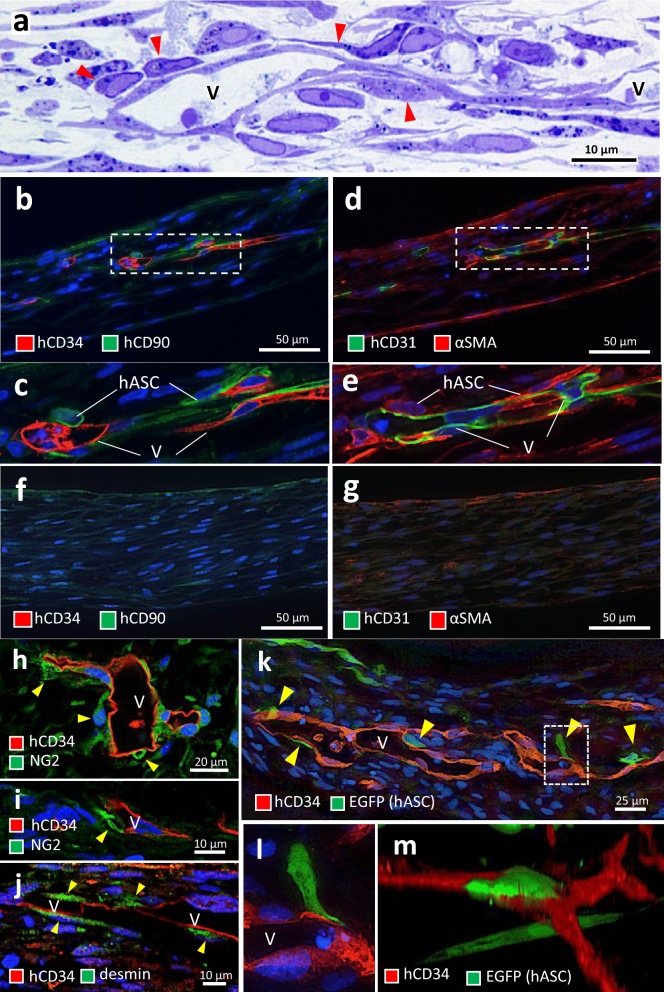
Perivascular differentiation of hASCs in ASCVT. (**a**) Highly magnified image of epon-embedded and sectioned ASCVT with toluidine blue staining. *V* vascular structures. Perivascularly localized hASCs are observed (red arrowheads). (**b**–**g**) Immunostaining for human CD34 (hCD34)/human CD90 (hCD90) (**b**,**c**,**f**) and human CD31 (hCD31)/αSMA (**d**–**g**). (**b**–**e**) ASCVT. Vascular structures (V) are immunostained for hCD34 and hCD31. Perivascularly localized hASCs show intense immunostaining for hCD90 and αSMA (**c**,**e**). (**f**,**g**) Artificial tissue constructed by hASCs without HUVECs. No vascular structures are found. A weak staining of hCD90 and αSMA is observed. (**h**,**i**) Perivascularly localized hASCs showing immunostaining for NG2 (yellow arrowheads). (**j**) Perivascularly localized hASCs showing immunostaining for desmin (yellow arrowheads). (**k**–**m**) Addition of EGFP-labeled hASCs to fibroblast-based vascular tissue (NHDFs:hASCs:HUVECs = 7:1:1). (**k**) Immunostaining of the paraffin section. hASCs with EGFP localized to perivascular area (yellow arrowheads). (**l**) Highly magnified image of hatched box in (**k**). EGFP-positive hASC connected to the vascular structure (V). (**m**) 3D-constructed confocal image. EGFP-positive hASC surrounded the vascular structure like a pericyte. The nuclei of the cells were visualized by DAPI (blue color). The images are representatives from two independent experiments.

In the high magnification image of ASCVT, we observed the perivascular hASCs adjacent to the basement of the vascular endothelial cells (Fig. 3a, arrowheads). On immunostaining, we found that the perivascular hASCs intensely expressed human CD90 (Fig. 3b,c), and αSMA (Fig. 3d,e). In contrast, these intense staining were undetectable in the artificial tissue constructed by hASCs without vascular network, although slight staining was observed over the tissue (Fig. 3f,g). Next, we examined immunostaining for pericyte markers such as NG2 and desmin, and found positive reaction in the perivascular hASCs (Fig. 3h–j).

To confirm the perivascular localization of hASCs with pericyte-like characteristics, we constructed a vascular tissue consisting of NHDFs, EGFP-labeled hASCs, and HUVECs in the ratio of 7:1:1. Four days after construction, EGFP-labeled hASCs localized around and adhered to the vascular structure (Fig. 3k, arrowheads, Fig. 3l,m).

**Figure 4:**
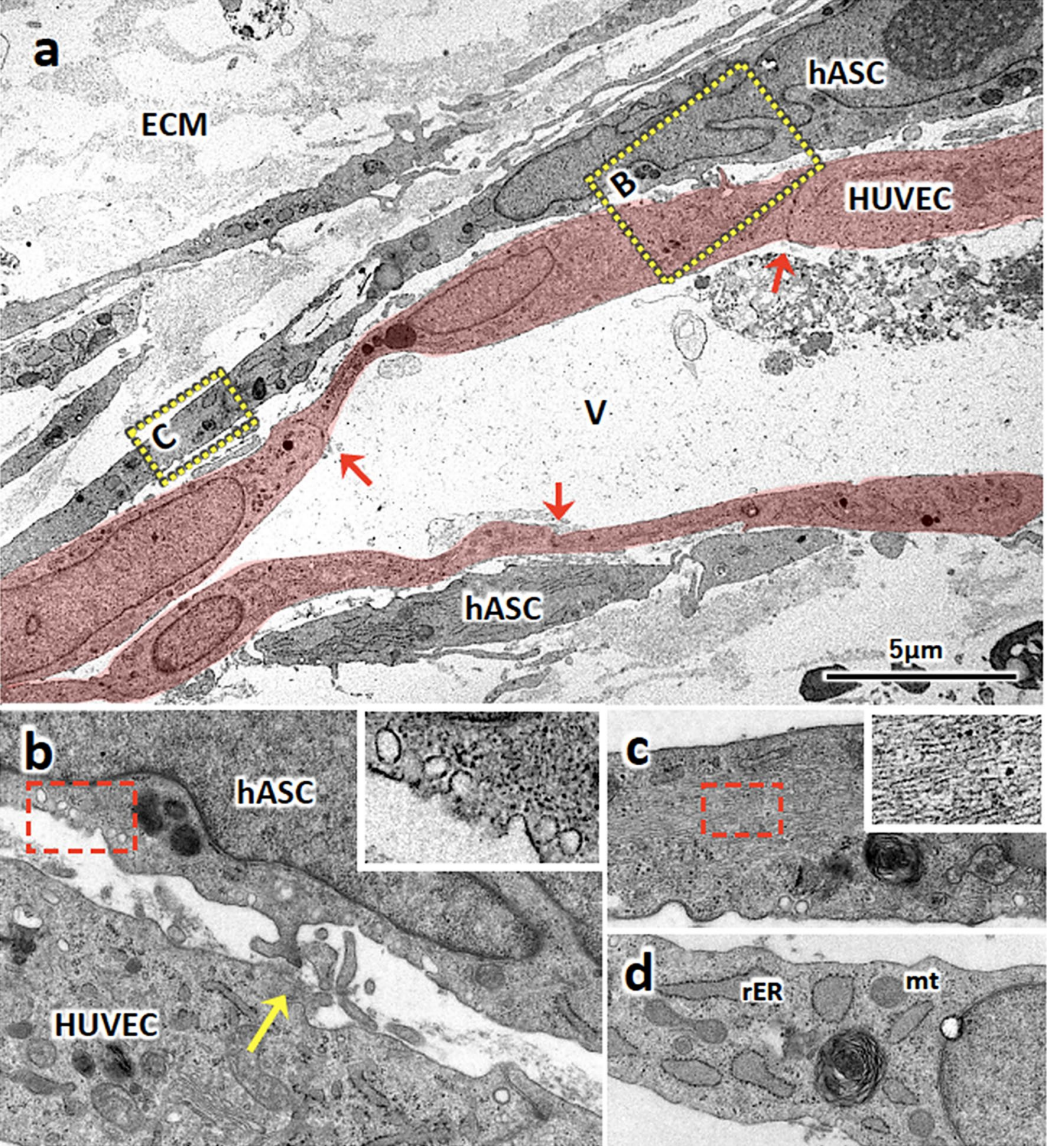
Ultrastructure of perivascularly localized hASCs in ASCVT. (**a**) Electron micrograph of ASCVT. V: vascular structure constructed by HUVECs (red-colored). Interendothelial connecting junctions are observed (red arrows). Perivascularly localized hASCs are also shown. ECM: extracellular matrix. (**b**) High magnified image of hatched box in (**a**). The projections from hASC interact with HUVEC, suggesting the formation of peg-socket structure (yellow arrow). Inset shows further high magnified image of red hatched box containing caveola at cytoplasmic membrane. (**c**) Another high magnified image of hatched box in (**a**). The cytoplasm of perivascular hASCs shows filamentous structure that is considered as presence of actin filaments. Inset shows further high magnified image of red hatched box on cytoplasm. (**d**) The image of hASCs locate far from vascular structures. The cytoplasm shows the fibroblast-like structure with abundant rough endoplasmic reticulum (rER) and mitochondria (mt). The images are representatives from three independent experiments.

We further observed the perivascular structure of ASCVT by transmission electron microscope (Fig. 4). The vascular structure constructed by HUVECs formed intercellular junctions (Fig. 4a, red arrow), likewise FbVT (Fig. S3g). The interaction between the perivascular hASCs and HUVECs was observed (Fig. 4b); yellow arrow indicates the projection of hASC connected to the basal part of HUVEC, suggesting the formation of peg-socket structure which is commonly found between endothelial cells and pericytes^30,31^. In addition, caveolae-like structures were observed in perivascular hASCs (Fig. 4b, inset). We also observed abundant intracytoplasmic filaments in these cells (Fig. 4c), which correspond to the immunostaining results of αSMA in Fig. 3d,e. In contrast, some hASCs apart from the vascular structure showed cytoplasmic ultrastructure resembling that of fibroblasts (Fig. 4d).

### Subcutaneous transplantation of ASCVT in nude mice

**Figure 5:**
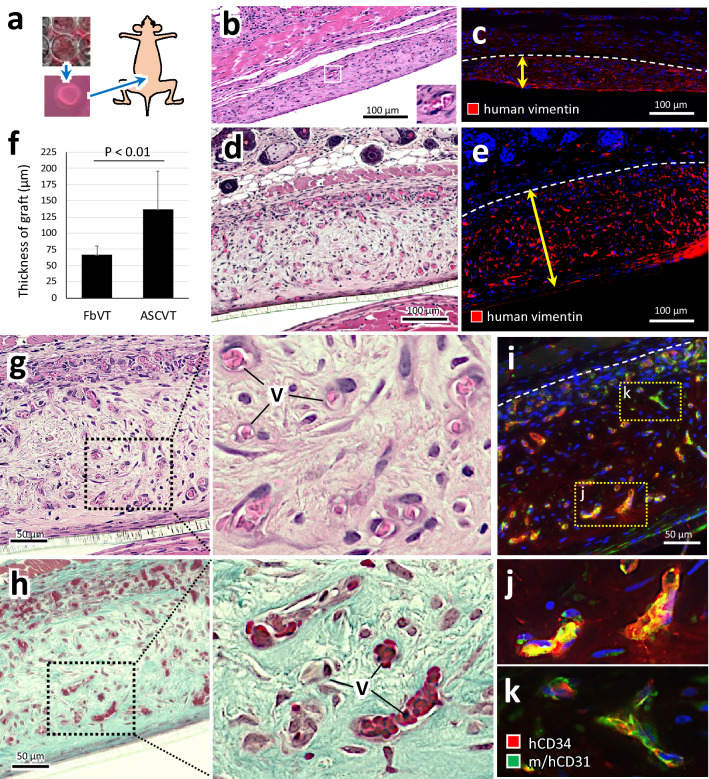
Subcutaneous transplantation of ASCVT to nude mice. (**a**) FbVT and ASCVT were cut out from the Transwell inserts and subcutaneously transplanted to back skin of nude mice. (**b**–**e**) Engrafted FbVT (**b**,**c**) and ASCVT (**d**,**e**) at 2 weeks after the transplantation. (**b**,**d**) HE staining; (**c**,**e**) immunostaining for human vimentin. The FbVT engrafts as connective tissue containing vascular networks with host blood (**b**, inset). The thickness of engrafted human-derived tissue is visualized by immunoreaction of human vimentin (**c**). The ASCVT also subcutaneously engrafted with vascular networks (**d**), and the thickness was higher than engrafted FbVT (**e**). (**f**) Quantitative comparison of the graft thickness between FbVT and ASCVT (N = 7). The ASCVT significantly shows higher thickness. (**g**–**k**) High magnified images of engrafted ASCVT. (**g**) HE staining. The graft contains the capillary- or small venule-like structures with host blood (V) and abundant stroma. (**h**) MassonGoldner staining for serial section of (**g**). The stroma stained for collagen fibers, indicating the construction of connective tissue. (**i**–**k**) Immunostaining by using antibodies for human CD34 (red) and mouse/human CD31 (green). The engrafted vessels derived from HUVECs are positive for both antibodies (**i**,**j**). At the peripheral area of the graft, the vessels with staining by antibodies for mouse/human CD31 and partial staining by antibodies for human CD34 are observed (**i**,**k**) suggesting the anastomotic region between host and graft circulation. The nuclei of the cells in the dark field images were visualized by DAPI (blue color). The images are representatives from three independent experiments.

The artificial vascular network tissues (FbVT and ASCVT) were cut out from Transwell inserts at 4 days after their fabrication and subcutaneously transplanted to the nude mice (Fig. 5a). After 2 weeks of transplantation, the engrafted tissues with host skin were collected, fixed and proceeded to tissue sectioning. The tissue sections were stained with hematoxylin and eosin (Fig. 5b,d) or immunostained for human vimentin (Fig. 5c,e). The engrafted FbVT had vascular structures that contained host erythrocytes (Fig. 5b, inset) as reported in a previous study^3^. Immunostaining for human vimentin indicated the area of the graft (Fig. 5c), and its average thickness was 66.95 µm (Fig. 5f). On the contrary, the engrafted ASCVT generated significantly thicker tissue than FbVT (Fig. 5e,f). The average thickness was 137.14 µm, approximately two times that of the FbVT (Fig. 5f). In the engrafted ASCVT, capillary- or small venule-like vascular structures were distributed in the abundant stroma (Fig. 5g) forming dense collagen fibers as shown by Masson–Goldner staining (Fig. 5h). The vascular structures contained the host erythrocytes (Fig. 5g,h, high magnification), which demonstrated the anastomosis to the host circulation system. The double immunostaining for human CD34 and mouse/human CD31 showed that the mosaic structure consists of human and mouse endothelial cells suggesting the anastomosed structures at periphery of the graft area (Fig. 5i–k).

These results demonstrated that ASCVT constructed by CP-CAM subcutaneously engrafted the connective tissue containing the vessels linked to host blood flow.

### Vascular structure of subcutaneously engrafted ASCVT in nude mice

**Figure 6:**
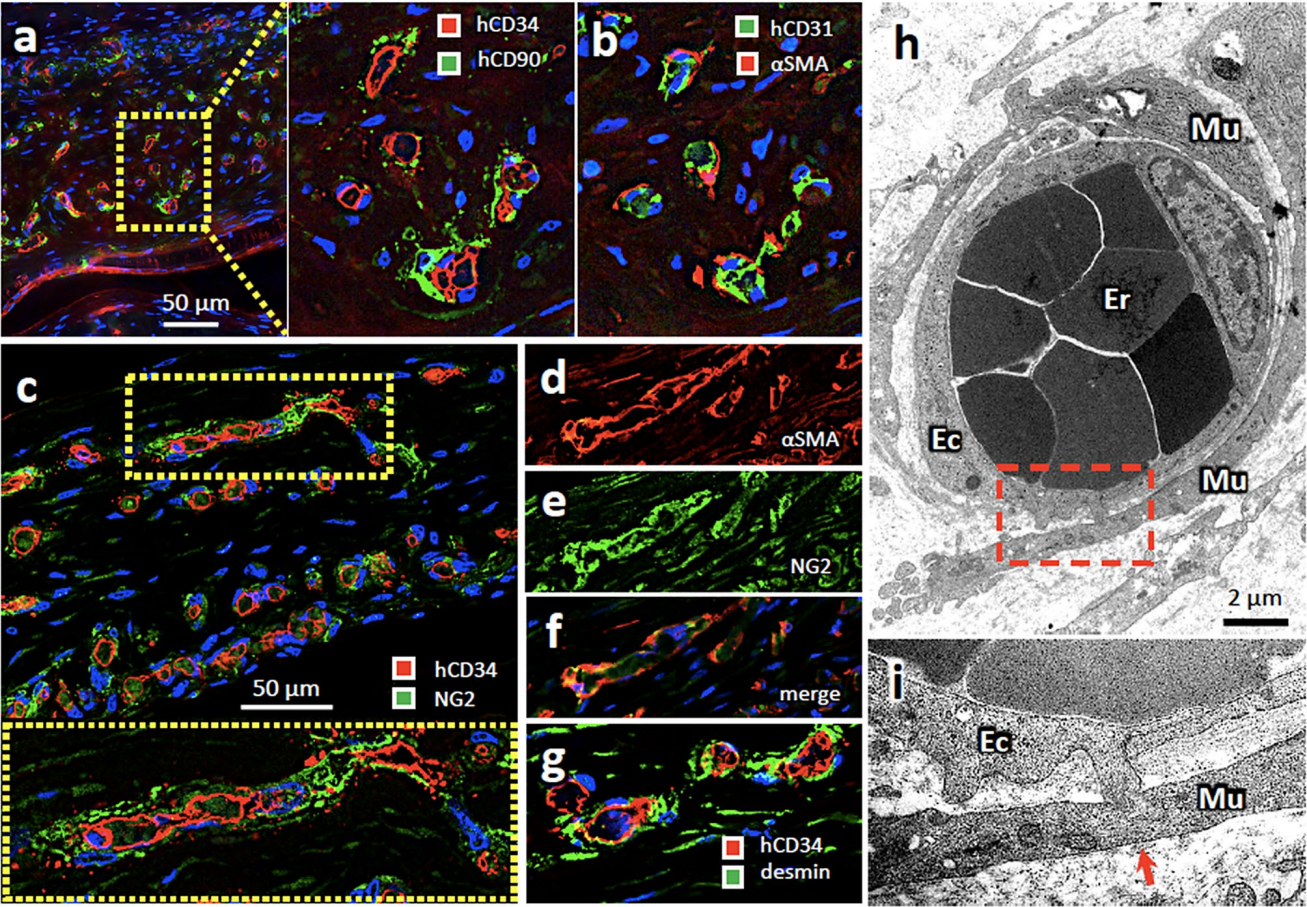
The vascular mural structure in the engrafted ASCVT at 2 weeks after the transplantation. (**a**) Immunostaining for human CD34 (hCD34) and human CD90 (hCD90). Almost every engrafted and hCD34-positive vessels surrounded by hCD90-positive mural cells that putatively derived from hASCs. (**b**) Immunostaining for human CD31 (hCD31) and αSMA in serial section of (**a**). The endothelial cells are positive for hCD31 in addition to hCD34. The mural cells are positive for αSMA. (**c**) Immunostaining for hCD34 and NG2. The mural cells are positive for NG2 that is a marker of pericytes and vascular smooth muscle cells. (**d**–**f**) Double immunostaining for αSMA and NG2. The merged image indicated coexistence of αSMA and NG2 in the mural cells derived from hASCs. (**g**) The presence of desmin, another marker of pericytes and smooth muscle cells, is confirmed in the mural cells. (**h**) Electron micrograph of the vessels in engrafted ASCVT. Er: erythrocytes, Ec: endothelial cells, Mu: mural cells. Stratifying mural cells surrounded the vessels. (**i**) High magnified image of hatched box in (**h**). Formation of peg-socket structure between endothelial cell and the mural cell is found (arrow). The nuclei of the cells in the dark field images were visualized by DAPI (blue color). The images are representatives from three independent experiments.

We further observed the mural structure of engrafted vessels in ASCVT by immunohistochemistry. After 2 weeks of transplantation, the human CD90-positive cells surrounded the vessels in the engrafted tissue (Fig. 6a) as well as αSMA-positive cells in the serial section (Fig. 6b). These mural cells were also positive for NG2 (Fig. 6c), corresponding to the presence of αSMA (Fig. 6d–f), and desmin (Fig. 6g). In the electron micrograph of the engrafted vessels, multiple mural cells localized around the endothelial tubules (Fig. 6h). Like the normal small vessels or capillaries, the basal part of endothelial cells and mural cells exhibited peg-socket structure in which the endothelial cells and mural cells can be connected (Fig. 6i, arrow)^30,31^.

We also subcutaneously transplanted the vascular tissue consisting of NHDFs, EGFP-labeled hASCs, and HUVECs in the ratio of 7:1:1, and analyzed the engrafted tissue (Fig. S7a–c). Almost every EGFP-labeled hASCs localized around human CD34-positive vessels (Fig. S7b,c), demonstrating that hASCs in the graft participated in constructing the mural structure in the artificial vessels.

**Figure 7:**
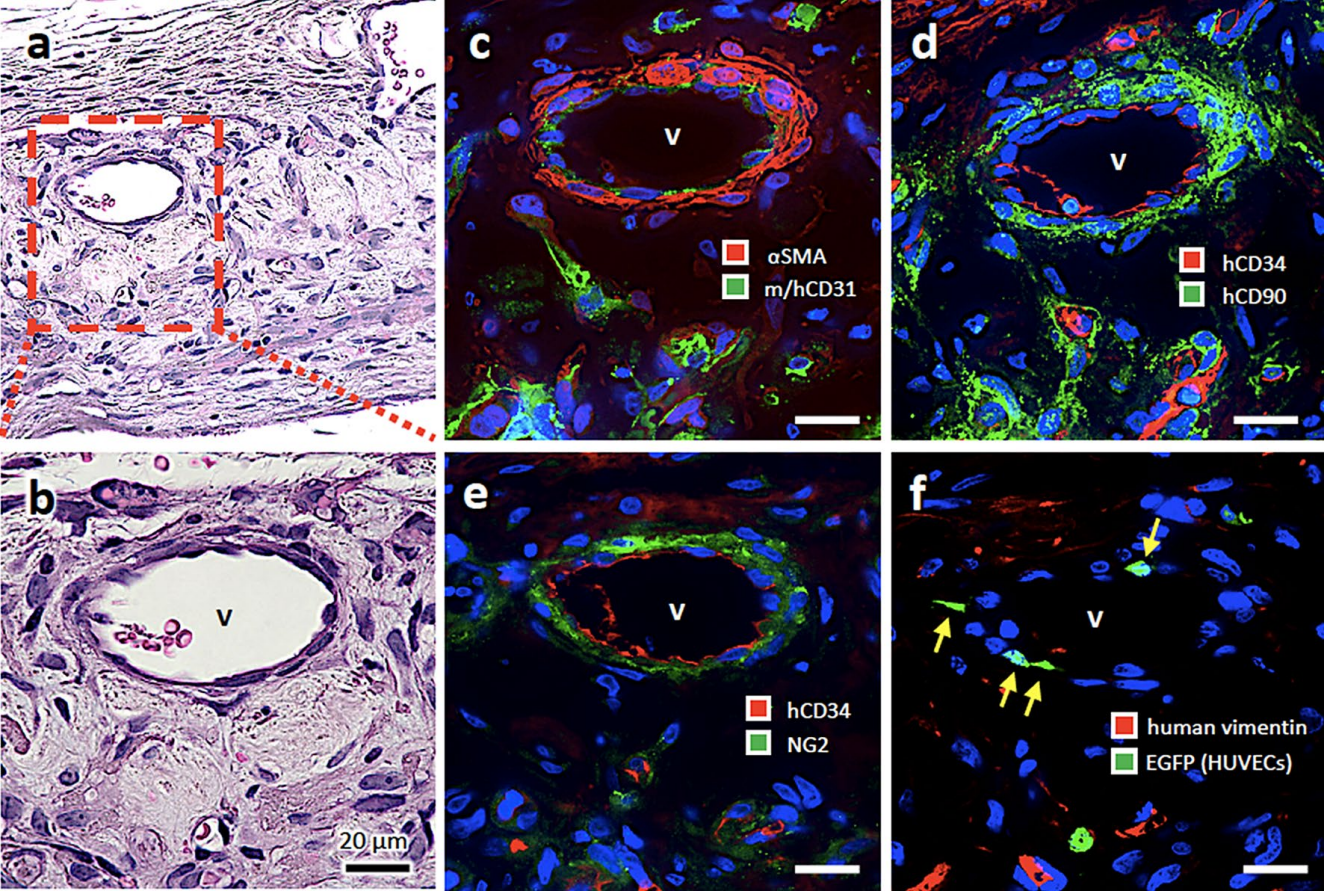
The blood vessels in the engrafted ASCVT at 4 weeks after the transplantation. (**a**–**f**) The engrafted ASCVT at 4 weeks after transplantation. The serial sections are used for each staining. (**a**,**b**) HE staining. Enlarged vein-like vessels (V) are found in the graft. (**c**–**f**) Immunostaining. The endothelium of vein-like vessel is positive for both mouse/human CD31 (m/hCD31) (**c**) and human CD34 (hCD34) (**d**,**e**). The mural cells show positive immunostaining for αSMA (**c**), human CD90 (hCD90) (**d**), and NGs (**e**), indicating that this structure derived from hASCs. (**f**) Immunostaining for human vimentin and EGFP. The engrafted ASCVT was constructed by using HUVECs labeled by EGFP. The endothelial cells showed EGFP-positive immunostaining (arrows), demonstrating that the vascular structures in ASCVT engrafted and constructed the vein-like vessels surrounded by mural cells. The nuclei of the cells in the dark field images were visualized by DAPI (blue color). The images are representatives from two independent experiments.

Moreover, in order to evaluate the long-term engraftment of ASCVT, we performed histological analysis of the graft 4 weeks after transplantation (Fig. 7). In this experiment, we used EGFP-labeled HUVECs for the fabrication of ASCVT. Although there was a replacement in some parts of the graft tissue by host tissue, we observed engrafted human blood vessels containing the host erythrocytes (Fig. 7a–f). Figure 7a,b demonstrate vein-like vessels with approximately 50 µm in diameter. These vessels showed positive immunostaining for human CD34 at endothelium. The vessels also showed staining for αSMA, human CD90 and NG2 at the mural structure constructed by 2–3 layers of mural cells (Fig. 7c–e). Supporting the growth of vessels, the expression of Ki67, a marker of cellular proliferation, was detected in endothelial cells and mural cells in the engrafted tissue (Fig. S8). Moreover, some of the endothelial cells showed EGFP-positive (Fig. 7f) proving that these cells derived from the artificial vessels constructed by HUVECs.

## Discussion

Efficient cryopreservation techniques for the stem cells or fabricated tissues will enable timesaving and convenient use in medical studies and clinical applications. Due to this, several cryopreservation techniques of the stem cells including ESC, iPSC, and mesenchymal stem/stromal cells, as well as cryopreservation of fabricated tissues such as cell sheets and spheroids have been reported^32–34^. In the present study, we developed CP-CAM for construction of transplantable artificial tissues with a process of cell cryopreservation. In our evaluation of cell cryopreservation media for CP-CAM, the CSFM which is a commercially available cell-freezing medium containing albumin, provided the most effective results of vascular network formation. Our designed cryopreservation medium (DMSO-albumin FM) containing recombinant human albumin also resulted in an efficient formation of vascular network by CP-CAM, suggesting that the albumin potentially plays a role in the preservation of vascular network-constructing capability of the frozen cells. Albumin has been used for cell or tissue cryopreservation as the stabilizer of cell surface proteins in previous studies^35,36^. Although the detail of mechanisms is unclear, it was considered that the albumin may stabilize the ECM nano-films at cell surface in cryopreservation. CSFM provided better results in recovery rate after thawing than DMSO-albumin FM (data not shown), suggesting that more factors in CSFM might support the preservation and tissue construction.

The CP-CAM is rapid and convenient for medical studies and clinical applications of artificial vascular tissue in regenerative medicine. After thawing of the cells from frozen stocks, the vascular tissue can be constructed within 4 days, that is the most timesaving compared to the other previous reports^5^. However, it should be noted that the recovery rates after cryopreservation were different among the tested cell types. Therefore, cryopreservation is preferable for some cell types with high recovery rates, combining with other cell types prepared without cryopreservation. In comparison among various conditions in CP-CAM, the recovery rates of the cells after thawing were not affected by cell concentrations and periods of cryopreservation. These results indicated the convenience of CP-CAM for experiments or therapeutic applications with flexible arrangement of the cells that are preserved in large scale for long-term periods.

By using CP-CAM, we constructed the artificial vascular tissue based on hASCs (ASCVT) and compared its structure to the tissue constructed based on human fibroblasts (FbVT). ASCVT had a thicker tissue structure and developed a denser vascular network than FbVT. ASCVT demonstrated abundant extracellular space between hASCs, and more Ki67-positive areas in the tissue compared to FbVT, suggesting that both higher extracellular matrix secretion and higher frequency of cell proliferation in ASCVT influenced on tissue thickness.

From the dense vascular network formation in ASCVT, we expected an abundant production of angiogenesis-related factors by 3D-cultured ASCs. As shown in the protein profile analysis of culture supernatant, we found a remarkable expression of six factors from 3D-cultured hASCs. Excluding for PF4 as an angiostatic factor^29^, the other five factors (VEGF-A, HGF, prolactin, MCP-1, and FGF-7) are pro-angiogenic/vasculogenic^24,26,37,38^. Although the promoted expression of VEGF-A and HGF in 3D cultivation of fibroblasts was reported in our previous study^9^, we found a more significant expression of these factors in 3D cultivation of hASCs. For other factors, prolactin induces angiogenesis through endothelial cell migration^26^, MCP-1 induces VEGF-A expression and tubular formation of endothelial cells, and FGF-7 is a potent inducer of angiogenesis/vasculogenesis^29,39^. From these results, 3D-cultured hASCs by CP-CAM were shown to promote the expression of angiogenesis-related factors and provided the inductive condition for artificial vascular network formation.

Since low oxygen condition had been shown in the artificial tissue fabricated by cell accumulation method^9^, we also examined the effect of hypoxic condition on the potent promotion of the factors from hASCs and confirmed that there was no correspondence of angiogenic factor expression profiles between hypoxic 2D cultivation and 3D cultivation of hASCs. This result suggested that some microenvironments induced in 3D culture of ASCs but not hypoxia are responsible for promoting the angiogenesis-related factors in ASCVT.

In the ASCVT, we found the pericyte-like characteristics in perivascular cells. In these cells, we observed an intense expression of human CD90 and pericyte markers such as αSMA, NG2, and desmin^40^, suggesting the differentiation of hASCs is possibly promoted by the interaction to HUVECs. The ASCs showing pericyte-like characteristics has been reported in the vascular morphogenesis by high-density cultivation of ASCs, which were considered to differentiate by the cellular interaction between heterotypic subpopulations^41^. Likewise, another study demonstrated that ASCs co-cultivated with endothelial cells in fibrin gel showed pericyte-like characteristics supporting and stabilizing the vascular structures^19^. In the adipose tissue, the ASCs distribute at perivascular areas^42^, and they are sometimes regarded as equivalent to pericytes because of the common markers and cellular characteristics^40,43^. Recent studies indicated that ASCs show pericytic phenotype promoted by NOTCH2 in the interaction between vascular endothelial cells^44^. Another study also indicated that hASCs act as pericytes and immunomodulatory functions^43^. In the present study, the hASCs showed pericyte-like differentiation under the presence of vascular structures. However, not all perivascular ASCs showed this phenomenon, indicating that the hASC subpopulation non-capable of pericyte-like differentiation may exist.

Along this differentiation, the pericyte-like cells showed intense CD90 expression. It was reported that the adventitial pericyte progenitor with MSC-like phenotype expressed CD90 with pericyte markers^45^. In addition, human lung tissue contained the pericyte-like subtype of MSCs with CD90 and pericyte marker expression^46^. Thus, it was considered that the same perivascular behavior of hASC subpopulations would be reproduced in our present study.

CD90 is one of the common markers for MSCs including ASCs^47^. Although its participation in self-renewal and differentiation of MSCs have been reported, the biological function of CD90 remains unclear^47^. Moraes et al. reported that knockdown of CD90 in MSC reduced the stemness guard of MSCs, enabling further differentiation when the specific stimuli are present^48^. For further analysis of perivascular MSC differentiation including the molecular mechanisms of CD90, the ASCVT has a high potential to be used as experimental tool.

The subcutaneous transplantation of ASCVT suggested their engraftment with an anastomosis to the host vessels and blood flow similar to the transplantation of FbVT^4^. The development of thicker tissues and dense vessel networks demonstrated prosperous tissue organization. After transplantation, ASCVT was found to have thicker structures than FbVT, with an abundant stroma forming dense collagen fibers which may be produced by hASCs, and high density of capillary-like vessel structures. The anastomotic structures of host and human vessels were also observed in the graft, suggesting the infiltration of host cells into the graft. Moreover, the Ki67-positive proliferating cells were detected in the hASCs especially at the perivascular area, and also in the endothelial cells. These would thicken the engrafted ASCVT under the microenvironment of host tissues.

To date, the induction of the tissue regeneration and angiogenesis by cellular transplantation of ASCs have been reported for therapeutic applications^49^. Our study showed that the scaffold-free 3D artificial vascular tissue based on hASCs could also promote both in vitro and in vivo tissue construction and vascular formation. The enhanced expression of pro-angiogenic factors shown in 3D cultivation of hASCs may contribute in these activities during in vivo processes.

Furthermore, we noticed the formation of perivascular mural structures by hASCs in the engrafted tissue. These structures exhibited pericyte- or vessel smooth muscle cell (SMC)-like characteristics with expression of NG2, αSMA, and desmin. Previously published data showed that different stimuli or co-culture methods induce ASCs to differentiate into pericytes or SMCs in vitro^41,44,50^. However, this differentiation in engrafted artificial tissue in vivo has never been reported. Our results clearly demonstrated that hASC-based perivascularly assembled and differentiated to form mural-like structures in engrafted ASCVT, suggesting formation of microcirculation with functional blood vessels. It is considered that enhanced pro-angiogenic activity and potency of hASCs in ASCVT developed in vitro would lead to the successful engraftment with structural maturation. Moreover, the maturation of engrafted vascular structure oriented from human cells was shown as development of blood vessels with large diameter in long-term periods after transplantation in our study. This result also suggested the development of functional vascular structure by transplantation of scaffold-free artificial vascular tissue. It has been reported that coverage of the vessels by mural structure and their interaction is necessary for vascular stabilization and further development^40,51^. Thus, the mural structure derived from hASC might promote the maturation of the blood vessels in transplanted tissue.

From our results by CP-CAM, the ASC-based vascular tissue using patient own cells can be used to enhance effective neovascularization. In this study, HUVECs were used as the source of vascular structure. For therapeutic applications, the vascular endothelial cells differentiated from the stem cells are possibly used instead of HUVECs^52,53^. The endothelial progenitor cells derived from peripheral blood or umbilical blood are also available^54,55^. We have already confirmed the potential of human umbilical blood-derived endothelial precursor cells by our artificial vascular tissue-constructing technique (unpublished data). Endothelial cells from these sources will enable to construct transplantable artificial vascular tissues as materials for regenerative medicine of ischemic diseases such as ischemic heart disease, ischemic stroke, and chronic limb ischemia^56–58^. For this purpose, further analysis and assessment of vascular function, engraftment persistence, and safety in clinical usage are needed.

In conclusion, CP-CAM is capable of rapid construction of pre-vascularized artificial tissue using hASCs. The ASCVT was subcutaneously transplantable and formed mature vascular networks alongside the engraftment. Our results demonstrated that cryopreserved human cells are able to construct the artificial transplantable tissue in short-term, and the patient’s own cells including ASCs are candidates for regenerative medicine. Furthermore, ASCVT will be a potent tool for studying perivascular differentiation of mesenchymal stem cells in vitro and in vivo models. The understanding of the intercellular communication and differentiation by using these models will lead to uncover the unknown mechanisms governing mesenchymal cell recruitment and differentiation alongside angiogenesis in development and regeneration.

## Methods

### Cell culture

Primary cultured NHDFs, neonatal hASCs and HUVECs were purchased from Lonza (Walkersville, MD). Each cell type was derived from single donor. NHDFs were cultured in Dulbecco’s modified Eagle medium (DMEM, Wako Pure Chemical, Osaka, Japan) containing 10% fetal bovine serum (FBS, SAFC Biosciences; Lenexa, KS) under 5% CO_2_ at 37 °C as described in our previous study^4^. For cultivation of HUVECs, Endothelial Growth Medium-2 (Lonza; Walkersville, MD) was used as described previously^3^. For cultivation of hASCs, Mesenchymal Stem Cell Growth Medium 2 (Takara Bio Inc., Kusatsu, Japan) was used. Transwell inserts with porous polyester bottom (pore size: 0.4 µm) for 24-well culture plate were purchased from CORNING Inc. (New York, NY).

### Animals

Female nude mice (BALB/cAJcl-nu) at 6 weeks of age were purchased from CLEA Japan, Inc. (Tokyo, Japan). All the animal experiments in this study were approved by the Animal Research Committee at Hirosaki University and were conducted according to the Guidelines for Animal Experimentation, Hirosaki University. Mice were maintained under controlled light (12-h light : dark cycle) and temperature (21 °C) conditions. The study was carried out in accordance with the ARRIVE guidelines.

### Construction of 3D artificial human vascular network tissue

Using a cell accumulation method by layer-by-layer cell coating technique, we constructed multi layered 3D tissue as previously described^8–10,60^. Briefly, cultured cells (NHDFs and hASCs within passage eight or HUVECs within passage six) were collected by trypsinization. The cells were suspended in 0.04 mg/mL bovine plasma-derived fibronectin [FN, Sigma-Aldrich (St. Louis, MO)] in 50 mM Tris–HCl buffer at pH 7.4, and incubated for 1 min with gentle rotation. Afterward, the cells were treated with 0.04 mg/mL of porcine skin gelatin [G, Sigma-Aldrich (St. Louis, MO)] in 50 mM Tris–HCl buffer. Nine steps of alternate FN and G treatment resulted in about 10 nm-thick coating of ECM nano-film on each cell type^8,9^.

The coated cells which were suspended in DMEM with 10% FBS were seeded on Transwell inserts. The inserts were set in 24-well cell culture plate supplemented with the same medium. To construct the vascular tissue, four bottom layers of NHDFs or hASCs, a single layer of HUVECs, and four top layers of NHDFs or hASCs were overlaid daily, and the tissues were continuously cultured in DMEM containing 10% FBS at 37 °C with 5% CO_2_ for 4 days. Alternatively, NHDFs or hASCs and HUVECs were mixed in the cell number ratio of 8:1, seeded on Transwell inserts, and cultured in DMEM containing 10% FBS at 37 °C with 5% CO2 for 5 days. In order to confirm the formation of the vascular network, the whole of fabricated 3D tissue was immunostained for human CD31 or human CD34 according to the method described below.

In this study, the artificial vascular tissues constructed by NHDFs/HUVECs and hASCs/HUVECs are termed FbVT and ASCVT, respectively. At least 10 times of ASCVT construction were performed for the analysis of tissue and transplantation in this study.

### Cryopreserved cell accumulation method (CP-CAM)

ECM nano-film-coated cells that were prepared as described in the former cell accumulation method above were cryopreserved using several cell freezing media as shown in Fig. S3a. After the coating, the cells were collected by centrifugation at 1000 rpm for 5 min, 15 °C, then resuspended in a cell freezing medium (CultureSure Freezing Medium; Fujifilm Wako, Osaka, Japan), termed as CSFM in this study, at the concentration of 5 × 10^5^–8 × 10^6^ cells/ml and placed on ice. The other four cryopreservation media: conventional cell freezing medium [DMEM containing 10% FBS and 10% dimethyl sulfoxide (DMSO)], CELLBANKER 1 (Takara Bio Inc.), STEMCELLBANKER (Takara Bio Inc.), and our originally designed cell freezing medium [DMEM containing 5 mg/ml of recombinant human albumin expressed in plants (Fujifilm Wako, Osaka, Japan) and 10% DMSO], termed as DMSO-albumin FM, were also examined. The cell suspensions were aliquoted in the cryovials, kept at − 80 °C overnight, and then stocked in liquid nitrogen.

In order to construct artificial tissue, the cryopreserved cells were thawed in the warm water at 37 °C for 2 min, resuspended in DMEM without the serum, and centrifuged at 1000 rpm for 5 min, 15 °C as illustrated in Fig. S3b. Then, the cells were washed with DMEM containing 10% fetal bovine serum and resuspended in the same medium with appropriate cell concentrations to fabricate the artificial tissue by the former cell accumulation method*.*

### Verification of the viable recovery rate after thawing of frozen cells in CP-CAM

The frozen cells (ECM nano-film-coated NHDFs, hASCs, and HUVECs) in a cryovial (1 ml) were thawed and the total cell number was counted. Then, the number was compared with that before freezing, and represent as % recovered rate;

$$Recovery\, rate\, (\%) = [cell\, number\, after\, thawing/cell\, number\, before\, freezing] \times 100.$$

The recovery rate of NHDFs with or without ECM nano-film coating and freezing by conventional medium was also assessed to verify the effectiveness of CP-CAM.

### Analysis of the angiogenesis-related factors profile

After cultivation of NHDFs and hASCs with suitable media, two-dimensional (2D) and 3D cultures were performed. For the 2D culture, 8 × 10^5^ of NHDFs or hASCs without ECM nano-film were seeded onto 3 cm-culture dishes with 2.3 ml of DMEM and incubated for 24 h under 5% CO_2_ at 37 °C. For the 3D culture, 8 × 10^5^ of NHDFs or hASCs with ECM nano-film were seeded on Transwell inserts and incubated with 2.3 ml of DMEM for 24 h under 5% CO_2_ at 37 °C. Then, the culture supernatants were collected from 2D/3D cultures (NHDFs or hASCs) and used to analyze the angiogenesis-related factors profile. The expression of 55 factors was analyzed using Proteome Profiler Human Angiogenesis Array Kit (R&D Systems) according to the manufacturer’s protocol. The quantitative analysis of detected factors was performed by using FIJI software (https://imagej.net/Fiji). The duplicated blots were obtained per factor, and their averaged scores were calculated.

### Transplantation of 3D artificial human vascular network tissue

Transplantation of the artificial vascular tissue was performed according to the method described in the previous studies^3,4^. Briefly, FbVT or ASCVT were collected from the Transwell inserts by cutting the bottom polyester membranes and kept in DMEM at 37 °C until transplantation into the subcutaneous tissue of nude mice. Under anesthesia, the dorsal skin of the mice was cut to a length of 1.5 cm, and the artificial tissues were then inserted subcutaneously, facing the artificial tissue up to the skin tissue. The wound was then immediately closed using silk sutures. For immunosuppression, cyclosporin (Neoral, Novartis, Rueil-Malmaison, France) was added to drinking water (120 mg/L), 1 week before and all along the engraftment period as previously reported^61^. After 2 weeks and 4 weeks of transplantation, the mice were euthanized, then the dorsal skin including the graft was collected for histological examination.

### EGFP labeling of hASCs and HUVECs by lentiviral transfection

For the fluorescence labeling of hASCs and HUVECs, GFP‐bearing CS‐CDF‐CG‐PRE lentiviral plasmid was used^59^. The experimental procedures were approved by the Genetically Modified Organisms Safety Committee of Hirosaki University (registration number: 16S001-1) and Tokyo Medical and Dental University (registration number: G2019-026C). To generate viral particles, the 293FT cells were co‐transfected with the pCMV‐VSV‐G‐RSV‐Rev expression plasmid and pCAG‐HIVgp packaging plasmid using Lipofectamine 2000 (11668019; Thermo Fisher Scientific). The lentiviral particles in the supernatants were collected 48 h after transfection, and infected to 5.0 × 10^4^ hASCs or HUVECs per well in 12‐well tissue culture plates.

EGFP-labeled hASCs were used for visualization of their perivascular localization in the artificial vascular tissue. The NHDFs, HUVECs, and EGFP-labeled hASCs were coated with ECM nano-film and seeded in the ratio of 7:1:1. At 4 days after the seeding, the tissues were fixed, embedded and immunostained for human CD34 and EGFP. These tissues were also subcutaneously transplanted to nude mice, and engrafted tissues at 2 weeks after transplantation were fixed and immunostained according to the method described below.

EGFP-labeled HUVECs were used for visualization of engrafted artificial human vascular structures in nude mice. The ASCVTs were constructed by using EGFP-labeled HUVECs and subcutaneously transplanted as described above. The engrafted tissues at 4 weeks after transplantation were fixed and immunostained for EGFP.

### Light and fluorescence microscopy

The fabricated artificial vascular tissues in the Transwell inserts were fixed with 4% paraformaldehyde in 0.1 M phosphate buffer (pH 7.4) for overnight at 4 °C and collected from the Transwell inserts by cutting the bottom polyester membranes. In order to achieve whole-mount immunostaining, the tissues were treated with 0.3% Triton X-100 in 0.1 M phosphate buffer at 4 °C for 3 days. Then, the tissues were stained according to the method described in our previous studies^3,4,10^. The specimens were observed using a confocal microscope Nikon C2 (Nikon, Tokyo, Japan). For the histological analysis, the fixed artificial tissues embedded in paraffin, and then 5 µm thick serial tissue sections were prepared. The engrafted tissues collected from mouse dorsal skin were also fixed in 4% paraformaldehyde in 0.1 M phosphate buffer (pH 7.4) and embedded in paraffin. Masson–Goldner staining was performed using the conventional method. After deparaffinization, the sections were treated with acid Fuchsin solution, phosphomolybdic acid-Orange G solution, and Light Green solution, respectively. Afterward, the sections were mounted using cover slips before observation. Immunohistochemistry was performed as follows. The deparaffinized sections were boiled twice in 10 mM citric acid (pH 6.0) using a microwave oven at 500 W for 5 min each for antigen retrieval. Then, the sections were treated with blocking solution [3% normal goat serum (Wako) in 0.1 M phosphate buffer (pH 7.4) containing 0.05% Tween 20] for 1 h at room temperature, and incubated with the primary antibodies overnight at 4 °C. For dark field microscopy, the sections were visualized by incubation with fluorescence-labeled secondary antibodies, goat anti-mouse IgG conjugated with Alexa-Fluor 594 or goat anti-rabbit IgG conjugated with Alexa-Fluor 488. The specimens were observed using a light microscope BX-50 (Olympus, Tokyo, Japan) or a fluorescence microscope BZ-X700 (Keyence, Tokyo, Japan).

### Transmission electron microscopy

The fixative containing 2.5% glutaraldehyde and 2% paraformaldehyde in 0.1 M phosphate buffer (pH 7.4) were used to fix the artificial vascular tissues and engrafted tissues at 4 °C. After that, the tissues were cut into 1 mm × 1 mm in size and post-fixed with 1% osmium tetroxide in 0.1 M phosphate buffer. Subsequently, the tissues were dehydrated and embedded in Epon 812 (Nisshin EM, Tokyo, Japan). Ultra-thin sections with 70 nm-thickness were prepared using an ultramicrotome (REICHERT ULTRACUT S, Leica, Wetzlar, Germany). After staining with 4% uranyl acetate and lead stain solution (Sigma Aldrich, St. Louis, MO), the tissues were observed under transmission electron microscope (JEM-1200, JEOL, Tokyo, Japan).

### Quantitative analysis of image data

Areas of human CD34-positive vascular network in immunofluorescent photographs were extracted by using Photoshop software (Adobe, San Jose, CA) and their total length, branches and areas were quantified by using Image J software (https://imagej.nih.gov/ij/) and Angiogenesis Analyzer (http://image.bio.methods.free.fr/ImageJ/?Angiogenesis-Analyzer-for-ImageJ&lang=en). Three-dimensional analysis of vascular network was also performed by using FIJI software (https://imagej.net/Fiji).

Ki67-positive areas in immunofluorescent photographs were extracted by using Photoshop software and quantified by using Image J software.

### Statistical analysis

The statistical comparisons between two independent groups of the data were performed by *t* tests. The normal distribution in all sample data was confirmed by the degree of skewness and kurtosis. Then, the equality of variances in two groups of the data was validated by *F* test. For the data with equal variances or unequal variances, Student's *t* test or Welche's *t* test were used, respectively. *P*-values of less than 0.05 were considered statistically significant.

## Supplementary Information

Supplementary Information.

## Data Availability

The data that support the findings of this study are available from the corresponding author (HS) upon request.
