# Supplementary material for: Construction of transplantable artificial vascular tissue based on adipose tissue-derived mesenchymal stromal cells by a cell coating and cryopreservation technique
Source: Sci Rep. 2021 Sep 9;11:17989. doi: 10.1038/s41598-021-97547-2 (PMC8429436; doi:10.1038/s41598-021-97547-2)
Supplement: Supplementary file 1 — Supplementary Information. [file 41598_2021_97547_MOESM1_ESM.pdf]

Construction of transplantable artificial vascular tissue based on adipose tissue-derived mesenchymal stromal cells by a cell coating and cryopreservation technique

Yoshiya Asano[1], Daisuke Okano[1], Michiya Matsusaki[2], Tetsuro Watabe[3], Yasuhiro Yoshimatsu[3,4], Mitsuru Akashi[5], and Hiroshi Shimoda[1,6*]

[1] Department of Neuroanatomy, Cell Biology and Histology, Graduate School of Medicine, Hirosaki University, Hirosaki, Japan

[2] Department of Applied Chemistry, Graduate School of Engineering, Osaka University, Osaka, Japan

[3] Department of Biochemistry, Graduate School of Medical and Dental Sciences, Tokyo Medical and Dental University (TMDU), Tokyo, Japan

[4] Division of Pharmacology, Graduate School of Medical and Dental Sciences, Niigata University, Niigata, Japan

[5] Building Block Science, Graduate School of Frontier Biosciences, Osaka University, Osaka, Japan

[6] Department of Anatomical Science, Graduate School of Medicine, Hirosaki University, Hirosaki, Japan

* Corresponding author: Hiroshi Shimoda, M.D., and Ph.D.

Telephone: +81-172-39-5004; Fax: +81-172-39-5006

E-mail: hshimoda@hirosaki-u.ac.jp

Supplementary information

1. Reagents

Bovine plasma-derived fibronectin (FN) and porcine skin gelatin (G) were purchased from Sigma-Aldrich (St. Louis, MO) and Wako Pure Chemical Industries, Ltd. (Osaka, Japan), respectively. CELLBANKER1 (cat. No. CB011) and STEM-CELLBANKER (cat. No. CB045) were purchased from Takara Bio Inc. (Kusatsu, Japan). CultureSure Freezing Medium (cat. No. 039-23511) was purchased from FUJIFILM Wako Pure Chemical (Tokyo, Japan). Mouse anti-human vimentin monoclonal antibody (cat. No. M7020), mouse anti-human CD31 monoclonal antibody (cat. No. M8023), mouse anti-human CD34 monoclonal antibody (cat. No. M7165) were purchased from DAKO (Glostrup, Denmark). Mouse anti-alpha smooth muscle actin (αSMA) monoclonal antibody (cat. No. ab40865), rabbit anti-human CD31 polyclonal antibody (cat. No. ab134168), and rabbit anti-mouse and human CD31 polyclonal antibody (cat. No. ab28364) were purchased from Abcam (Austin, TX). Rabbit anti-human CD90 polyclonal antibody (cat. No. AC-0054RUO) was purchased from EPITOMICS (Burlingame, CA). Rabbit anti-NG2 polyclonal antibody (cat. No. AB5320) was purchased from Merck Millipore (Darmstadt, Germany). Rabbit Ki67 polyclonal antibody (cat. No. 27309-1-AP) and rabbit anti-desmin polyclonal antibody (cat. No.16520-1-AP) were purchased from Proteintech (Rosemont, IL). Alexa Fluor 594-conjugated goat anti-mouse IgG (cat. No. A11032) and Alexa Fluor 488-conjugated goat anti-rabbit IgG (cat. No. A11034) were purchased from Invitrogen (Carlsbad, CA). DAPI solution was purchased from Wako Pure Chemical (Osaka, Japan). Protein Profiler Human Angiogenesis Array Kit (cat No. ARY007) was purchased from R & D Systems (Minneapolis, MN).

2. Evaluation of the cell freezing media and conditions for CP-CAM

In order to adopt cryopreservation technique to the cell accumulation methods, we examined the effect of several cell freezing media on vessel network formation in artificial vascular tissue constructed by NHDFs and HUVECs (FbVT) (Fig. S1). In the control tissue (constructed by cell accumulation method without cryopreservation), the HUVECs formed human CD34-positive vascular network (Fig. S1a) as similar to findings in previous studies [3, 4, 10]. However, cryopreservation using conventional cell

freezing medium [Dulbecco's modified Eagle medium (DMEM) containing 10% DMSO and 10% FBS] resulted in the formation of a funicular structure by HUVECs without vascular network (Fig. S1b). Moreover, the vascular network could not be formed when the cells were cryopreserved in two commercially available media, CELLBANKER1 (with serum) and STEM-CELLBANKER (without serum) as shown in Fig. S1c and d, respectively. On the other hand, the ECM nano-film-coated cells formed vascular network similar to the control group after cryopreservation in another commercially available medium, CultureSure Freezing Medium without serum but containing bovine serum-derived albumin (termed as CSFM in this study) (Fig. S1e). Due to the CSFM contains DMSO and albumin, we then designed our original freezing medium containing DMSO and recombinant human albumin (refers to DMSO-albumin FM as described in Materials and Methods) and examined its cryopreserved effect on vascular network formation. The cells cryopreserved using this medium were able to form human CD34-positive vascular network as shown in Fig. S1f. Although both CSFM and DMSO-albumin FM achieved the vascular formation, we obtained higher yields of living cells after thawing of cryopreserved stocks in CSFM (data not shown). Therefore, we further used CSFM for the CP-CAM in this study.

Fig. S2a showed the recovery rate of NHDFs, hASCs, and HUVECs after cryopreservation. The recovery rate of NHDFs was 75.3% in the condition of non-coating and usage of conventional cell freezing medium (10% DMSO and 10% FBS in DMEM). An addition of ECM nano-film coating did not affect the recovery rate (76.4%). On the other hand, the condition of ECM nano-film coating and usage of CSFM provided significantly higher recovery rate (87.4%) than the other conditions. In this condition, the recovery rate of hASCs and HUVECs was lower than that of NHDFs (79.9% and 69.0%, respectively). The results suggested that the recovery rates is different depending on cell types.

We further evaluated the recovery rates of NHDFs, hASCs, and HUVECs in CP-CAM by using CSFM focusing on the cell concentration and the length of the cryopreservation period (Fig. S2b and c). From the results, we found that the recovery rates of each cell type were not significantly affected by the cell concentrations and cryopreservation periods.

3. Artificial 3D vascular tissue constructed by CP-CAM

We analyzed the effect of CP-CAM on the vascular structures in FbVT prepared by ECM nano film-coated NHDFs and HUVECs without and with cryopreservation (refer to usual cell accumulation method and CP-CAM, respectively). On comparing these artificial tissues, we observed no significant differences between the vascular network in FbVT constructed by usual cell accumulation method and CP-CAM (Fig. S3c and d). The tubular structures were formed in FbVT constructed by CP-CAM as shown in Fig. S3e, V. We observed a few necrotic cells with swollen shape in the tissue suggesting a slight influence of cryopreservation (Fig. S3e, black arrows).

In the transmission electron micrograph, the tissue consisted of fibroblasts, vascular structures constructed by HUVECs, and extracellular matrix (Fig. S3f, Fb, V, and ECM, respectively). Formation of intercellular junctions between overlapping endothelial cells and basal lamina surrounding vascular structures were also found (Fig. S3 g, red arrow). These features corresponded to those in our previous study in which the vascular tissue was fabricated by usual cell accumulation method [3].

For further convenience, we also tested the cryopreservation mixing of the ECM nano-films coated NHDFs and HUVECs in ratio of 8:1. The seeding of thawed cells in nine layers on Transwell insert also resulted in the formation of vascular networks within four days as shown in Fig. S4.

These results demonstrated that the artificial vascular tissues can be rapidly constructed by CP-CAM, with almost equivalent structure as usual cell accumulation method previously reported.

4. Supplementary figures

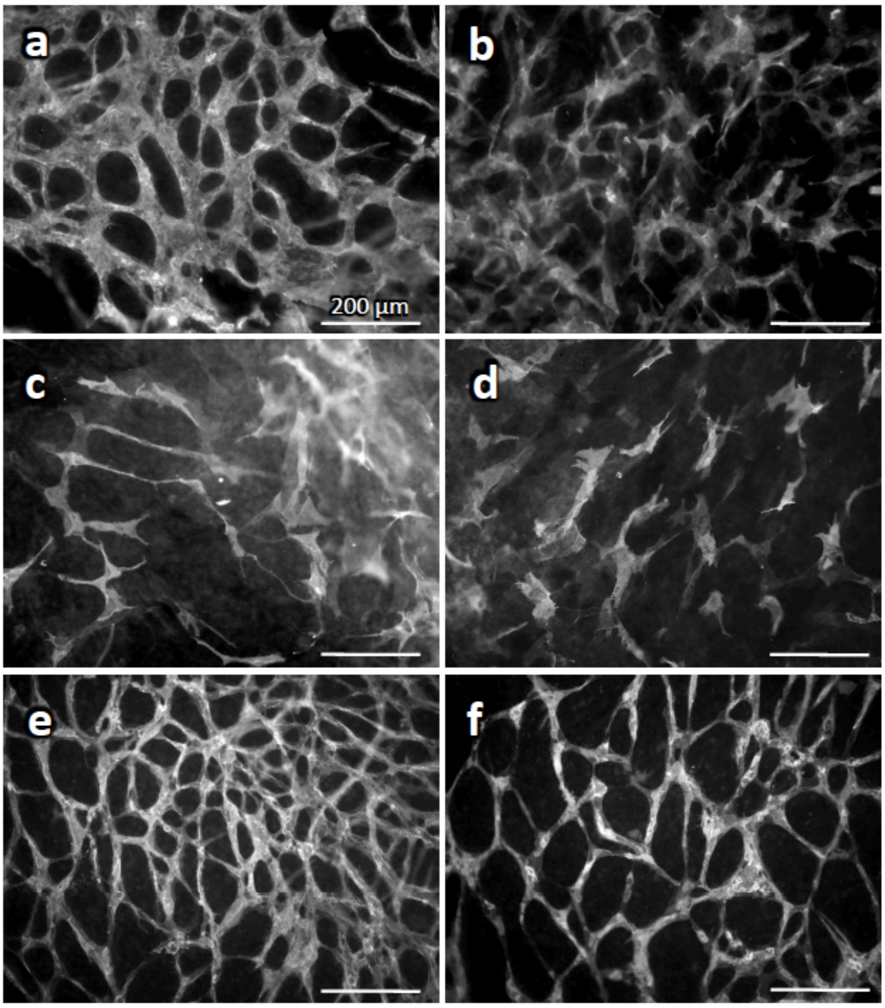

Figure S1: Comparison of cell freezing media for cryopreserved cell accumulation method (CP-CAM)

Artificial vascular tissues constructed by NHDFs and HUVECs, termed as FbVT, were used for evaluation of cell freezing media for CP-CAM. The vascular networks are visualized by immunostaining for human CD34. **a**: Without cryopreservation of ECM nano-film-coated cells (control). **b**: DMEM containing 10% DMSO and 10% FBS. **c**: CELLBAMKER 1 (Takara Bio Inc.). **d**: STEMCELLBANKER (Takara Bio Inc.). **e**: CultureSure Freezing Medium (CSFM, Fujifilm Wako). **f**: DMEM containing 10% DMSO and 5 mg / ml of recombinant human albumin (DMSO-albumin FM).

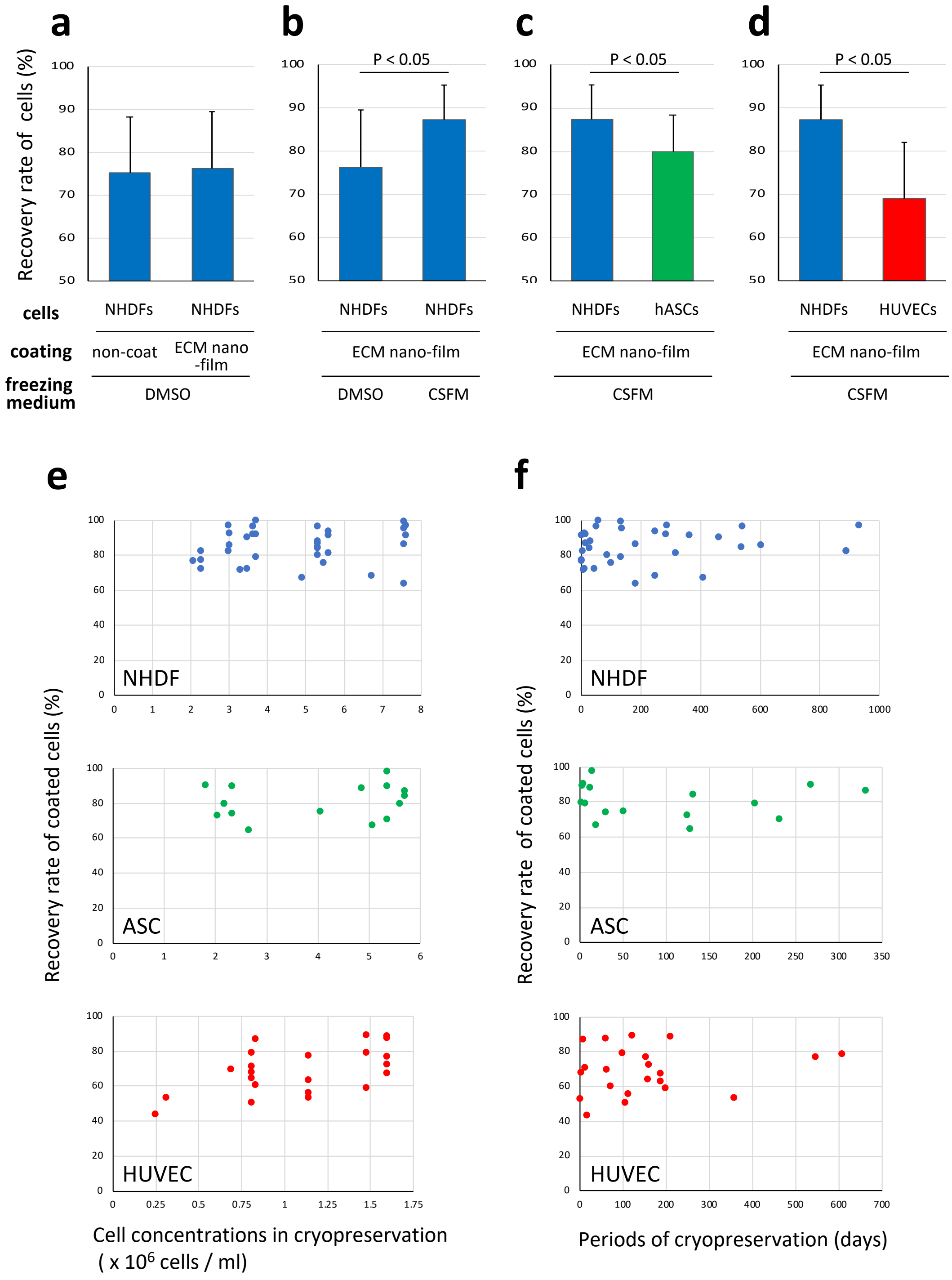

a
b
c
d
Recovery rate of cells (%)
100
90
80
70
60
50
P < 0.05
cells
NHDFs
NHDFs
hASCs
HUVECs
coating
non-coat
ECM nano -film
ECM nano-film
freezing medium
DMSO
CSFM
e
f
Recovery rate of coated cells (%)
NHDF
ASC
HUVEC
Cell concentrations in cryopreservation (x 10⁶ cells / ml)
Periods of cryopreservation (days)

Fig. S2

Figure S2: Recovery rates after thawing of cryopreserved cells by using CSFM in CP-CAM

To evaluate the recovery of living cells after cryopreservation by using CSFM, the thawed cells were stained with 2% trypan blue in phosphate buffered saline (pH 7.3), and the cells with trypan blue-exclusion were counted as living cells. The ratio of living cell number after thawing to that before cryopreservation were compared between several cell concentrations and preserved periods in liquid nitrogen. **a-d**: The comparison of recovery rates in various conditions and cell types. **a**: NHDFs frozen in conventional cell freezing medium (DMEM containing 10 % DMSO / 10 % FBS, termed as DMSO) without ECM nano-film coating (non-coat, N = 5) and those with the coating (ECM nano-film, N = 7). **b**: ECM nano-film-coated NHDFs frozen with conventional cell freezing medium (DMSO, N=7) and with CSFM (N = 22). **c**: ECM nano-film-coated NHDFs (N = 22) and hASCs (N = 20) frozen with CSFM. **d**: ECM nano-film-coated NHDFs (N = 22) and HUVECs (N = 22) frozen with CSFM. In case of ECM nano-film-coated NHDFs, significant increase of the recovery rate is shown in cryopreservation with CSFM compared to those with DMEM containing 10 % DMSO / 10 % FBS. Recovery rate of hASCs and HUVECs is lower than that of NHDFs. **e**: The recovery rates of ECM nano-film coated cells after cryopreservation in CSFM at various cell concentrations. The recovery rates did not significantly alter even at high cell concentrations (8×10^6 cells/ml for NHDFs). **f**: The recovery rates of ECM nano-film coated cells after cryopreservation in CSFM at various cryopreservation periods. Long-term preservation did not affect the recovery rates after thawing (up to 930 days for NHDFs).

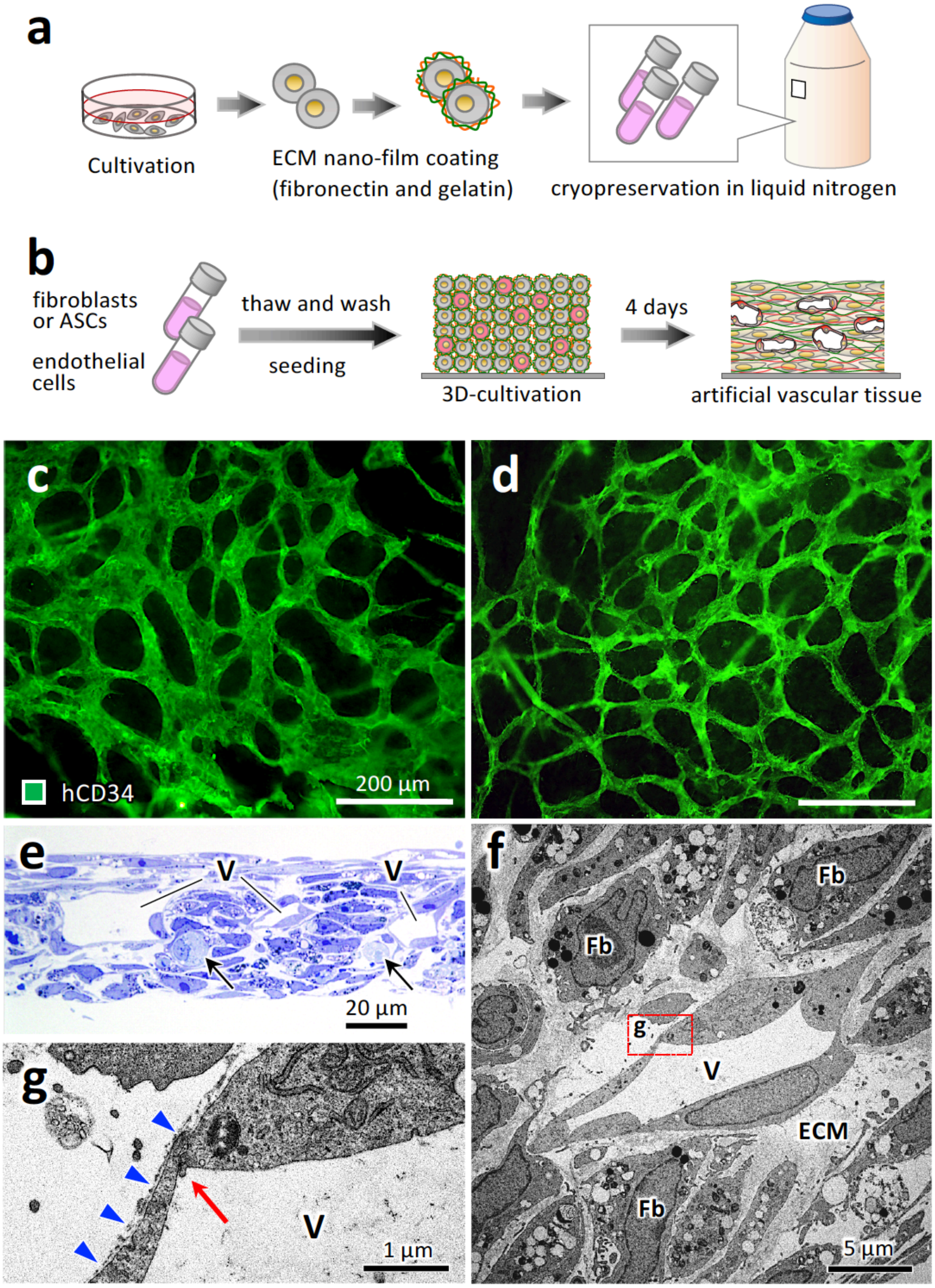
a
Cultivation
ECM nano-film coating
(fibronectin and gelatin)
cryopreservation in liquid nitrogen
b
fibroblasts
or ASCs
endothelial
cells
thaw and wash
seeding
3D-cultivation
4 days
artificial vascular tissue
c
hCD34
200 µm
d
e
V
V
20 µm
f
Fb
Fb
g
V
ECM
Fb
5 µm
g
V
1 µm

Figure S3: Construction of artificial 3D vascular tissue by CP-CAM

a and **b**: The process of CP-CAM. The detailed procedure is described in Methods. **a**: Briefly, after the cultivation, the cells are coated with ECM nano-films and cryopreserved in liquid nitrogen for long-term storage. **b**: For tissue construction, cryopreserved cells are thawed, washed, and used for tissue construction. In case of the vascular tissue, fibroblasts or ASCs are mixed with the vascular endothelial cells in appropriate ratio and seeded on Transwell inserts. The artificial tissue with vascular networks is constructed within four days after the seeding. **c - f**: Histological images of FbVT. **c** and **d**: Human CD34 (hCD34)-positive vascular network in the tissue constructed by cell accumulation method without cryopreservation (**c**) and that constructed by CP-CAM (**d**). **e**: The vascular tissue constructed by CP-CAM. The tissue was cut and stained by toluidine blue. V: vascular structures constructed by HUVECs. Arrows: a few of necrotic cells are observed in the tissue. **f**: Electron micrograph of the tissue. Fb: fibroblast. ECM: extracellular matrix. **g**: The enlarged image of red box in f. The vascular structure is constructed by interendothelial connecting junction (red arrow). The basal lamina at the outside of the vascular structure is also found (arrowheads).

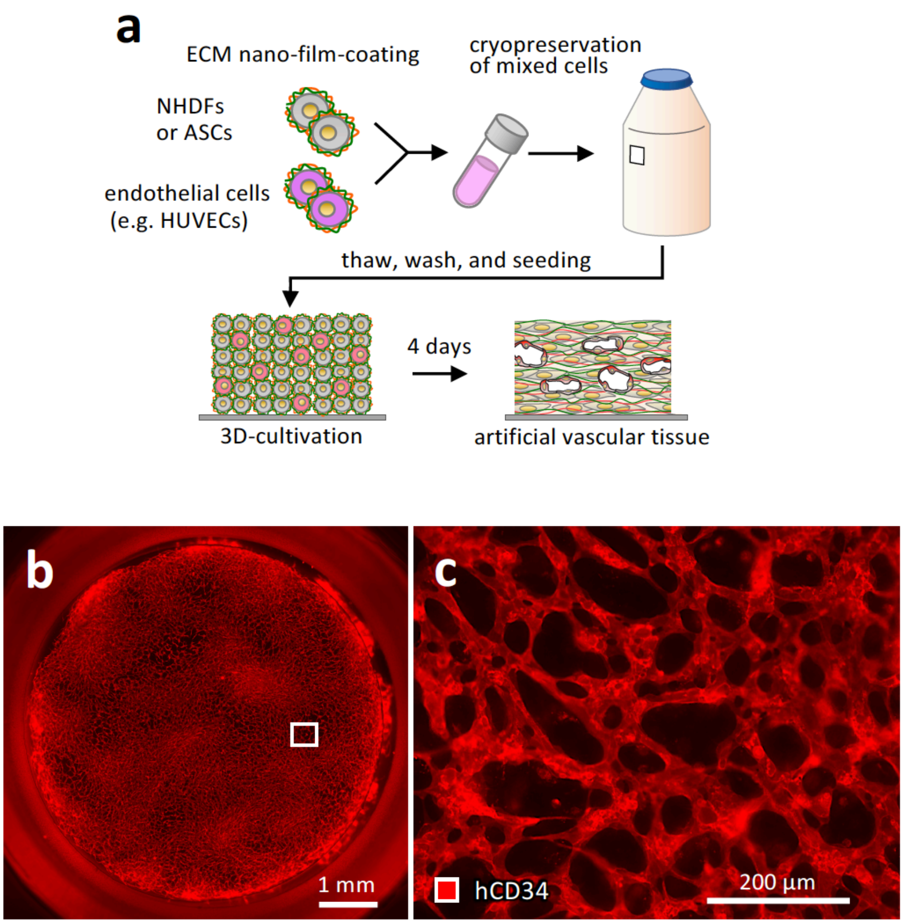

Figure S4: Rapid construction of artificial vascular tissue by CP-CAM with mixed cells.

a: Schematic representation of method for rapid construction of artificial vascular tissue by CP-CAM with mixed cells. The ECM nano-film-coated NHDFs or ASCs are mixed with coated endothelial cells before cryopreservation. After thawing and washing, the cells are seeded to construct vascular tissue. **b** and **c**: Artificial vascular tissue constructed by mixed NHDFs and HUVECs (8:1). The vascular network with human CD34-positive immunostaining is constructed within four days after seeding.

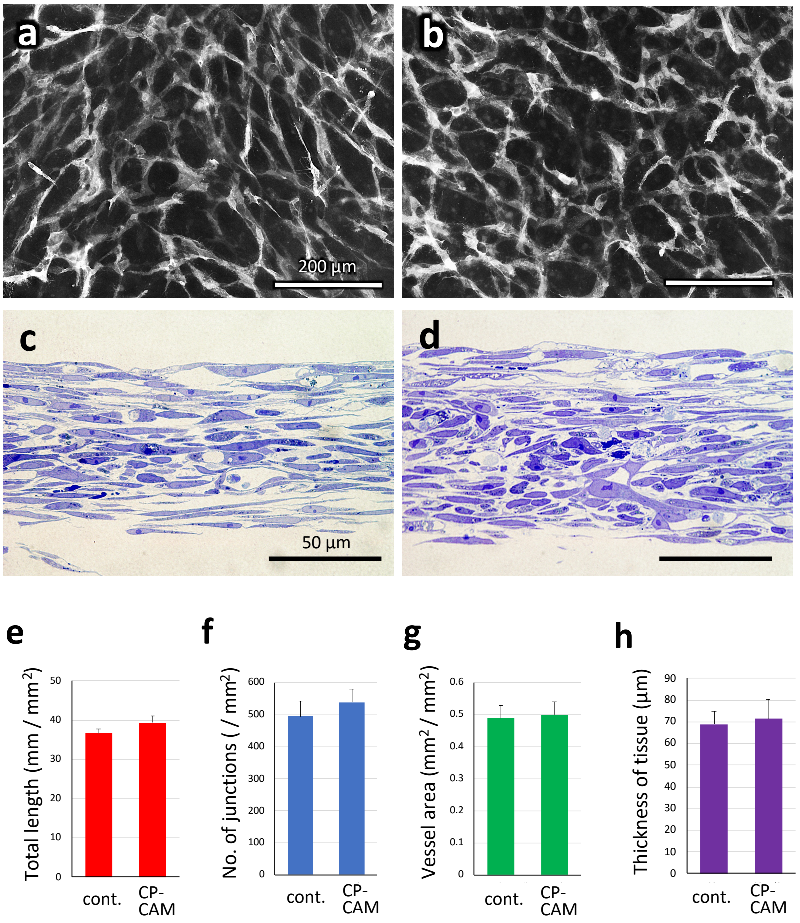

Figure S5: Comparison of ASCVTs constructed by usual cell accumulation method and CP-CAM

The influence of cell freezing on ASCVT construction is evaluated by comparison the tissue structure between usual cell accumulation method (control) and that with cryopreservation of coated cells (CP-CAM). **a** and **b**: Histological images of ASCVTs. The vascular networks are visualized by immunostaining for human CD34. No remarkable difference is observed between the control (**a**) and CP-CAM (**b**). The quantitative comparison of the vascular network structures in total length (**e**), number of junctions (**f**), and vessel area (**g**) also demonstrate that there is no significant difference between control and CP-CAM. **c** and **d**: The sections of epon-embedded ASCVTs with toluidine blue staining. No difference in the tissue structures is observed between the control (**c**) and CP-CAM (**d**). There is also no significant difference in the thickness of the tissues (**h**). e-g: N = 6, h: N = 4.

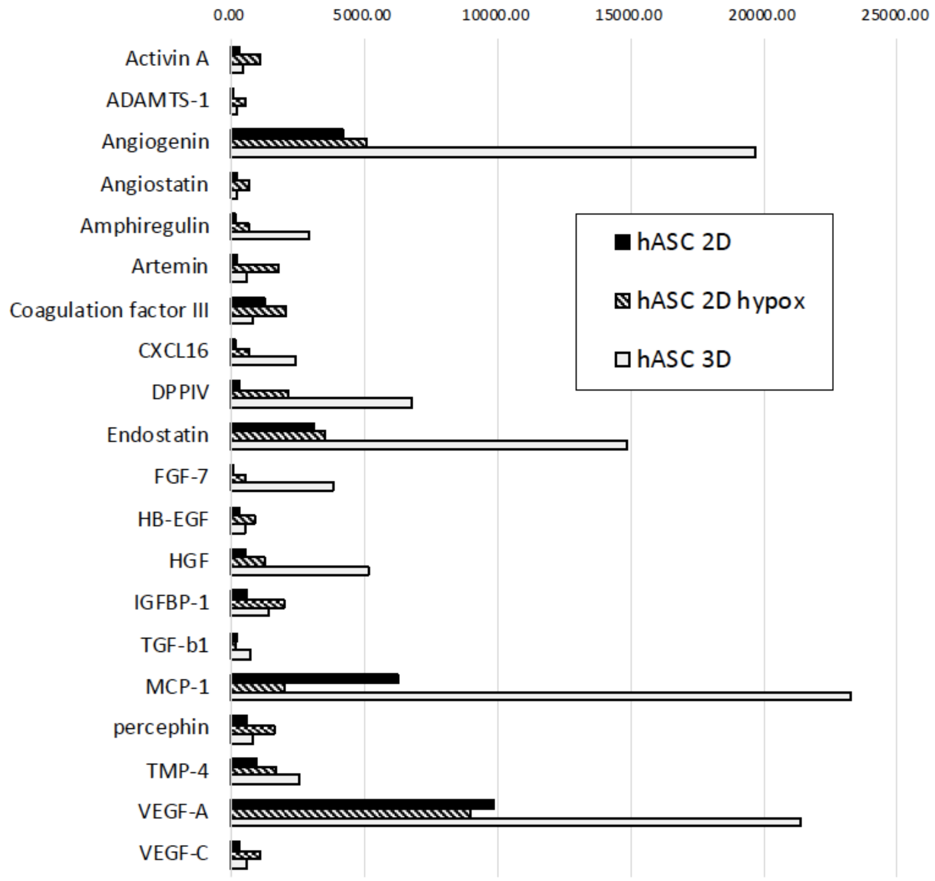

Figure S6: Profile of angiogenesis-related factors in culture supernatants collected from 2D-cultivated hASCs under hypoxic condition

The culture supernatants of 2D-cultivated hASCs under hypoxic condition (5% CO_2 and 1% O_2) were analyzed by using Proteome Profiler Human Angiogenesis Array Kit (R&D Systems), and compared to those of 2D- or 3D-cultivated hASCs under normal culture condition (5% CO_2 and 21% O_2). The profile of 2D-cultured hASCs under hypoxic condition (striped bars) does not corresponded to that of 3D-cultured hASCs (white bars), indicating that the increase of angiogenesis-related factors in 3D-cultured hASCs is probably promoted by some other factors in 3D microenvironment, rather than hypoxic condition.

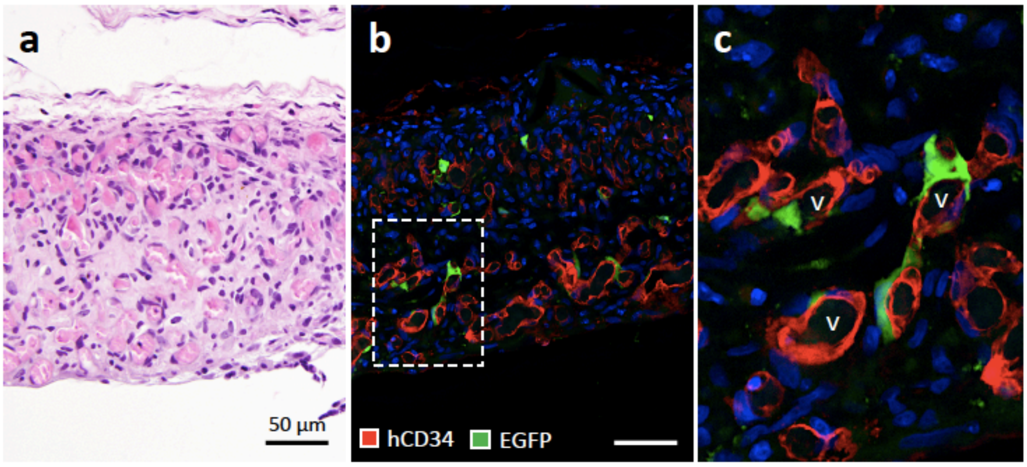

Figure S7: Perivascularly localization of EGFP-labeled hASCs in the engrafted vascular tissue

Fibroblast-based vascular tissue with addition of EGFP-labeled hASCs (NHDFs : hASCs : HUVECs = 7: 1: 1) was subcutaneously transplanted in nude mouse. **a**: HE staining of engrafted vascular tissue at two weeks after transplantation. **b** and **c**: Immunostaining for human CD34 (hCD34) and EGFP. The EGFP-positive cells perivascularly localized. This result supports that the mural structures observed in the engrafted ASCVT shown in Fig. 6 and 7 are derived from hASCs.

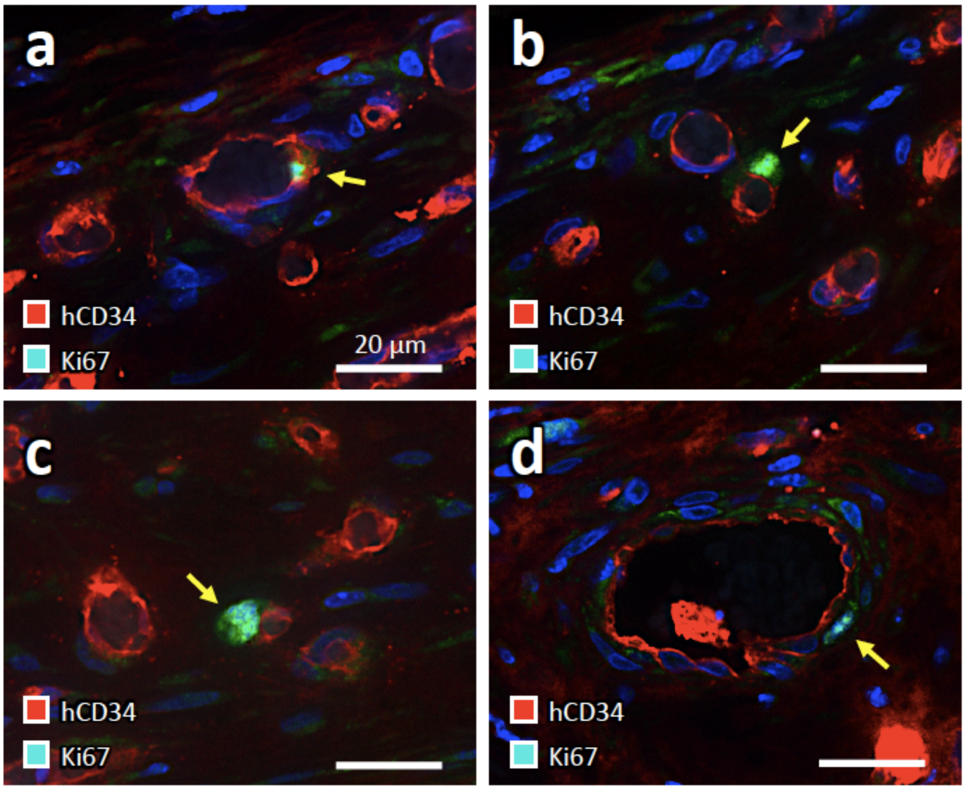

Figure S8: Detection of cellular proliferation marker in the engrafted vessels of ASCVT

Cellular proliferation in the engrafted ASCVT was detected by immunostaining for Ki67. The arrow shows Ki67-positive cells. Engrafted vessels are visualized by immunostaining for human CD34 (hCD34). **a - c**: engrafted ASCVT at two weeks after transplantation. **a**: Ki67-positive endothelial cell is detected. **b** and **c**: Ki67 was detected in perivascular cells those are the mural cells putatively derived from hASC. **d**: engrafted ASCVT at four weeks after transplantation. Ki67-positive cell is observed in the mural structure.
